# Determination of Rare Earth Element Isotopic Compositions Using Sample-Standard Bracketing and Double-Spike Approaches

**DOI:** 10.1021/acsearthspacechem.3c00172

**Published:** 2023-11-06

**Authors:** Justin Y. Hu, Francois L. H. Tissot, Reika Yokochi, Thomas J. Ireland, Nicolas Dauphas, Helen M. Williams

**Affiliations:** † Origins Laboratory, Department of the Geophysical Sciences and Enrico Fermi Institute, The University of Chicago, Chicago, Illinois 60637, United States; ○ Department of Earth Sciences, University of Cambridge, Cambridge CB2 3EQ, United Kindgom; ∥ The Isotoparium, Division of Geological and Planetary Sciences, California Institute of Technology, 1200 E. California Blvd., Pasadena, California 91125, United States; ⊥ Department of Earth and Environment, Boston University, 685 Commonwealth Avenue, Boston, Massachusetts 02215, United States; § Department of Earth Sciences, University of Cambridge, Cambridge CB2 3EQ, United Kingdom

**Keywords:** Rare earth elements, mass-dependent isotopic fractionation, isotopes, double-spike, DSCII, FPLC

## Abstract

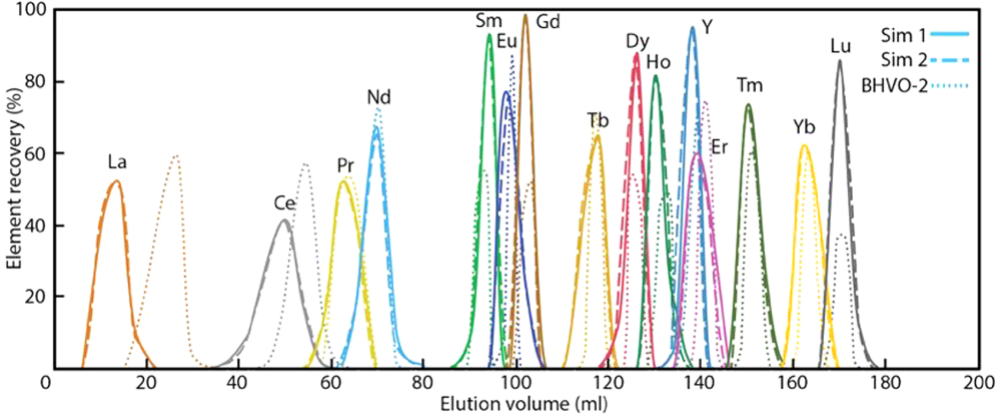

Rare earth elements (REEs) have been found to have numerous uses to trace geological and cosmochemical processes through analyses of elemental patterns, radioactive decay, nucleosynthetic anomalies, and cosmogenic effects. Stable isotopic fractionation is one aspect of REE geochemistry that has been seldom studied, with most publications focusing on the development of analytical methodologies for individual REEs, and most applications concerning terrestrial igneous rocks. In this study, we present a method to systematically analyze stable isotopic fractionations of 8 REEs, including Ce, Nd, Sm, Eu, Gd, Dy, Er, and Yb, using sample-standard bracketing (SSB) and double-spike (DS) approaches. All REEs are separated and purified using a fluoropolymer pneumatic liquid chromatography (FPLC) system. We introduce procedures for identifying and correcting some isobaric interferences in double-spike data reduction. Several geostandards, including igneous rocks and sediments, are analyzed using SSB and DS methods. The results indicate that REE isotopic fractionation in igneous processes is limited, except for Eu. Other REEs can still be isotopically fractionated by low-temperature processes and kinetic effects at a high temperature.

## 1 Introduction

Rare earth elements (REEs) comprise 15 lanthanides, including La, Ce, Pr, Nd, Pm (decays with a short half-life), Sm, Eu, Gd, Tb, Dy, Ho, Er, Tm, Yb, and Lu, as well as Sc and Y. Due to the steady decrease of their ionic radius with increasing nucleus mass, the chemical behavior of REEs varies smoothly as a function of their atomic number. The abundances of REEs have therefore been normalized to reference materials, typically CI chondrites^1−3^ and Post Archean Australia Shales (PAAS^3−5^), to track numerous geochemical and cosmochemical processes such as assessing degree of partial melting, fractional crystallization, and magma mixing in igneous processes, tracing the source of sediments, and providing clues on past ocean chemistry and circulation.^6^ Radiogenic isotope systems including ^138^La–^138^Ce, ^146^Sm–^142^Nd, ^147^Sm–^143^Nd, and ^176^Lu–^176^Hf are widely used for dating purposes and for tracing water circulation (e.g., refs (7−10)). Some REEs are affected by cosmogenic effects in rocks exposed to cosmic rays at the surface of airless bodies, and these effects have been used to monitor neutron-capture effects and understand the regolith history of the Moon, Mars, and Vesta (e.g., refs (8 and 11−15)).

The most substantial REE stable isotopic fractionations reported so far are in the group II calcium–aluminum-rich inclusions (CAIs) analyzed in Hu et al.^16^ (e.g., −2.3‰/amu for Gd, −3.1‰/amu for Dy, and −3.6‰/amu for Er). The substantial negative isotopic fractionations of REEs in group II CAIs are primarily controlled by kinetic effects associated with evaporation and condensation in the solar nebula.

Hu et al.^17^ analyzed the force constants of ^151^Eu and ^161^Dy using the synchrotron technique of nuclear resonance inelastic X-ray scattering (NRIXS) on a variety of synthetic compounds and silicate glasses. They extrapolated the measured force constants to other REEs to predict the equilibrium mass-dependent fractionations (MDFs) of most REEs (particularly the vibrational contribution of the crystal lattice) and found that in typical terrestrial high-temperature processes the MDFs are negligible. The application of equilibrium MDFs for most REEs is largely restricted to low-temperature environments (e.g., ref (18)) or high-temperature environments involving kinetic effects such as diffusion.^19−24^ For example, the rapid growth of clinopyroxene phenocrysts during melt interaction with reactive porous flow can induce measurable Nd stable isotopic fractionation.^25^

Europium stands out among other REEs as the nuclear field shift (NFS) effect seems to dominate equilibrium isotope fractionation associated with redox processes.^26^ Compared to MDF induced by the lattice vibration (written below as MDF for simplicity), NFS scales as the reciprocal of temperature in K (1/*T*) rather than 1/*T*^2^, meaning that equilibrium Eu isotopic fractionation could remain significant at igneous temperatures.^27^ Cerium isotopes can also be affected by NFS effects that are opposite to those imparted by MDF.^25^

Expanding the database of REE stable isotopic fractionation by investigating extraterrestrial samples, low-temperature samples, and high-temperature samples influenced by NFS and kinetic effects would improve our understanding of the geochemical behavior of REEs in diverse environments. Reported REE isotope fractionations are still relatively limited (Ce,^18,28,29^ Nd,^25,30−32^ Sm,^16,33,34^ Eu,^16,27,35−37^ Gd,^16^ Dy,^16^ Er,^16,38^ and Yb^16,38^), with most publications focused on developing analytical methods for one or two REEs, and the samples analyzed are primarily geostandards and igneous rocks.

Here, we present methodologies to purify and analyze the stable isotopic fractionations of 8 REEs (Ce, Nd, Sm, Eu, Gd, Dy, Er, and Yb). The other two REEs with two isotopes, La and Lu, were not analyzed because the abundance contrast between their isotopes is large (0.09% and 99.91% for ^138^La and ^139^La, 97.41% and 2.59% for ^175^Lu and ^176^Lu). All REEs are separated from each other and purified through a fluoropolymer pneumatic liquid chromatography (FPLC) system developed at the Origins Laboratory of The University of Chicago. We compare REE isotopic analyses that use (i) sample-standard bracketing (SSB) for all the REEs, (ii) double-spike (DS) approaches wherever possible (Ce, Nd, Sm, Gd, Dy, and Yb), and (iii) DS approaches for REEs not adjacent to each other (Ce, Sm, Dy, and Yb) to avoid isobaric interferences resulting from multielement spikes.

We extended the mathematical treatment presented in Hu and Dauphas^39^ to derive a formula to correct laboratory-induced mass fractionation that takes into account both isotopic anomalies and isobaric interferences in the DS approach. We introduce a method (double-spike correction for isobaric interference, DSCII) that uses 5 or more measurable isotopes to detect and correct for some isobaric interferences. The application of DSCII is illustrated in the DS reduction of Nd, Sm, Gd, and Yb. Elements with 5 or more measurable isotopes such as Ca, Ti, Mo, and Ba can benefit from DSCII for evaluating data quality and detecting and correcting potential isobaric interferences.

A set of geostandards including igneous (basalts, andesites, and granites) and metamorphic (schist) rocks and sediments (limestone, ferromanganese deposits, and iron formation) are analyzed for REE isotopes using both SSB and DS methods. The results are compared to published values and used to evaluate REE isotopic fractionation in nature.

## 2 Method

### 2.1 Reference Material Preparation

High purity (>99.99%) REE oxide powders in the forms of Nd_2_O_3_, Sm_2_O_3_, Eu_2_O_3_, Gd_2_O_3_, Dy_2_O_3_, Er_2_O_3_, and Yb_2_O_3_ were purchased from High Purity Metal Specialists (ESPI) as reference materials. High purity (>99.995%) Ce_2_(CO_3_)_3_ was purchased from Sigma-Aldrich as Ce reference materials. Approximately 50 to 200 mg of REE oxide or carbonate powder is weighed and dissolved in 50 g of 3 mol/L HNO_3_ to prepare 1000 ppm stock solutions. The stock solutions are further diluted in 0.3 mol/L HNO_3_ to 1 ppm for isotope measurements. The REE powder and stock solutions are available upon request (named OL-REE series^16^).

### 2.2 Double-Spike Preparation

The double-spike technique was used to measure the isotopes of Ce, Nd, Sm, Gd, Dy, and Yb. The enriched isotopes of Ce (^136^Ce and ^138^Ce), Nd (^145^Nd and ^146^Nd), Sm (^147^Sm and ^149^Sm), Gd (^155^Gd and ^157^Gd), Dy (^161^Dy and ^163^Dy), and Yb (^171^Yb and ^174^Yb) were procured from Oak Ridge National Laboratories (ORNL). The enriched isotopes of each REE were dissolved from oxide powder (Ln_2_O_3_) in 3 mol/L HNO_3_ and then mixed according to the optimal DS compositions determined in Rudge et al.^40^ The mixtures were used as stock solutions and further diluted to 1 ppm by using 3 mol/L HNO_3_ for calibration and isotopic analysis.

### 2.3 Nonspiked Sample-Standard Bracketing

The stable isotopic fractionations of the samples are presented as deviations in per mil per atomic mass unit (‰/amu) relative to the standards prepared from reference materials,
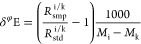
1where the superscript φ denotes the δ notation on a per amu basis, *M*_i_ and *M*_j_ are the mass numbers of isotopes ^i^E and ^k^E, and *R*_smp_^i/k^ and *R*_std_^i/k^ are the isotopic ratios of the sample and standard. For isotopic measurements conducted by SSB, the measurements of samples were interspersed by those of standards to account for the substantial but relatively stable temporal variations in instrumental mass bias in MC-ICP-MS,
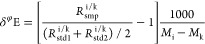
2where *R*_std1_^i/k^ and *R*_std2_^i/k^ are the measured isotope ratios before and after the sample measurement. The isotopic ratios used in eq 2 for all the REEs measured are ^142^Ce/^140^Ce, ^148^Nd/^144^Nd, ^147^Sm/^152^Sm, ^153^Eu/^151^Eu, ^160^Gd/^156^Gd, ^164^Dy/^162^Dy, ^167^Er/^168^Er, and ^174^Yb/^172^Yb, respectively.

The reported δ^φ^E values were calculated based on 3 to 12 standard-sample-standard bracketings. The confidence intervals (CIs) of the isotopic fractionations are reported as 95% CI using the student *t*-value and the variability of the sample δ^φ^E values.

### 2.4 Double-Spike Data Reduction

The double-spike technique (e.g., refs (40−42)) has been used since 1963 to correct for instrumental mass bias.^43^ This technique was later adopted in isotopic analyses by MC-ICP-MS for elements with 4 or more measurable isotopes (see ref (44) for an adaptation of the DS technique to three-isotope systems). A spike with a distinct isotopic composition from the target element is added to the sample in the early stage of the chemical procedure (e.g., ideally before digestion). The sample and spike are homogenized so that spike and sample atoms in the mixture experience the same chemical process and isotopic fractionation. Assuming that mass fractionation follows the exponential law, the isotopic ratios of the spike-sample mixture measured on the mass spectrometer can be written as,
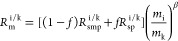
3where *R*_m_^i/k^, *R*_smp_^i/k^, and *R*_sp_^i/k^ are the measured, sample, and spike ratios of isotope ^i^E and ^k^E, *f* is the proportion of ^i^E from the spike in the spike-sample mixture, β is the instrumental mass bias, and *m*_i_ and *m*_k_ are the atomic mass of isotopes ^i^E and ^k^E. The sample isotope ratio *R*_smp_^i/k^ can be related to the standard isotope ratio *R*_smp_^i/k^ through the natural fractionation factor α.
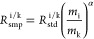
4

Rewriting eq 3 by substituting *R*_smp_^i/k^ in eq 4, we have
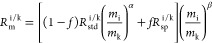
5

If the ratios of standard *R*_std_^i/k^ and spike *R*_sp_^i/k^ are known, the unknowns *f*, α, and β can be determined by solving a set of 3 equations corresponding to eq 5 applied to all isotopic ratios produced by combining 4 isotopes. The absolute isotopic ratios of the samples can be calculated from the natural fractionation factor α in eq 4.

For elements with more than 4 isotopes, one can calculate more than 3 ratios, so there are more equations than unknowns. In that case, one can estimate *f*, α, and β by minimizing the following quantity
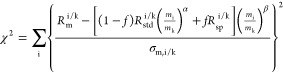
6where σ_m,i/k_ is the standard deviation of the measured ^i^E/^k^E ratio.^45^ The isotopes used for DS minimization of Nd, Sm, Gd, Dy, and Yb isotopic analyses are ^142^Nd–^144^Nd–^145^Nd–^146^Nd–^148^Nd–^150^Nd, ^147^Sm–^148^Sm–^149^Sm–^152^Sm–^154^Sm, ^155^Gd–^156^Gd–^157^Gd–^158^Gd–^160^Gd, ^161^Dy–^162^Dy–^163^Dy–^164^Dy, and ^171^Yb–^172^Yb–^173^Yb–^174^Yb–^176^Yb.

Implementation of the DS technique on MC-ICPMS usually involved bracketing spike-sample mixed solutions by spike-standard mixtures before and after the sample measurements (DS bracketing, DSB; e.g., ref (38)). The isotopic ratios of the sample and standard after the application of the DS reduction procedure were used in eq 2 to obtain the isotope fractionation of the sample relative to the standard. The δ^φ^E values were calculated based on 9 to 12 standard-sample-standard bracketings with 95% CI using the student *t*-value.

Application of the DS spike data reduction procedure requires knowledge of the isotopic composition of the spike (eq 5). The spike isotopic composition of each REE was determined by counter-spiking, which involves analyzing a mixture of reference material and spike and solving for a set of 3 equations modified from eq 5 where the positions of *R*_std_^i/k^ and *R*_sp_^i/k^ were swapped. For the standard isotopic ratios, we measured the pure OL-REE standards and applied internal normalizations to correct for instrumental mass bias by fixing ^140^Ce/^142^Ce = 7.9471, ^148^Nd/^144^Nd = 0.241579, ^148^Sm/^150^Sm = 1.523370, ^158^Gd/^156^Gd = 1.213485, ^164^Dy/^162^Dy = 1.109323, ^167^Er/^168^Er, and ^174^Yb/^172^Yb = 1.477200.

As part of the counterspike procedure, we also evaluated the optimal mixing ratio of the sample and spike by analyzing a suite of sample-spike mixtures with varying mixing ratios from approximately 10% spike to 90% spike (the percentages here refer to the fraction of spike atoms in the spike-sample mixture). The 95% confidence interval of the isotopic composition of the spike was calculated for each mixture based on the cycle-level variations in each measurement. The compositions of the spikes and the optimal spike proportions are presented in Table 1. The tolerance ranges of the mixing ratios were calculated by identifying the mixing ratios where the values overlapped with the optimal value within their confidence intervals.

**Table 1:** Isotope Compositions of Spikes and Spike-Sample Mixtures^a^

Ce	proportion	136	138	140	142
Ce-136 spike	0.4435	**0.5054**	0.0034	0.4579	0.0333
Ce-138 spike	0.5565	0.0019	**0.2600**	0.6990	0.0384
Double-spike	0.4517	0.2252	0.1462	0.5921	0.0361
Standard	0.5483	0.0019	0.0025	0.8844	0.1112
Optimal mixture		0.1027	0.0674	0.7524	0.0773

Nd	proportion	142	143	144	145	146	148	150
Nd-145 spike	0.4152	0.0092	0.0070	0.0255	**0.9173**	0.0367	0.0028	0.0015
Nd-146 spike	0.5848	0.0042	0.0029	0.0068	0.0064	**0.9763**	0.0024	0.0010
Double-spike	0.7352	0.0063	0.0046	0.0146	0.3846	0.5862	0.0026	0.0012
Standard	0.2648	0.2717	0.1218	0.2380	0.0829	0.1718	0.0575	0.0563
Optimal mixture		0.0766	0.0357	0.0737	0.3048	0.4765	0.0170	0.0157

Sm	proportion	144	147	148	149	150	152	154
Sm-147 spike	0.6296	0.0005	**0.9830**	0.0085	0.0036	0.0011	0.0021	0.0012
Sm-149 spike	0.3704	0.0003	0.0038	0.0079	**0.9768**	0.0056	0.0039	0.0017
Double-spike	0.6422	0.0004	0.6203	0.0083	0.3641	0.0028	0.0028	0.0014
Standard	0.3578	0.0307	0.1500	0.1124	0.1382	0.0738	0.2674	0.2275
Optimal mixture		0.0113	0.4520	0.0455	0.2833	0.0282	0.0975	0.0823

Gd	proportion	152	154	155	156	157	158	160
Gd-155 spike	0.6136	0.0001	0.0023	**0.9431**	0.0285	0.0102	0.0105	0.0053
Gd-157 spike	0.3864	0.0005	0.0031	0.0402	0.0560	**0.7971**	0.0809	0.0228
Double-spike	0.6036	0.0003	0.0026	0.5943	0.0391	0.3142	0.0377	0.0121
Standard	0.3964	0.0020	0.0218	0.1480	0.2047	0.1565	0.2484	0.2186
Optimal mixture		0.0009	0.0102	0.4173	0.1048	0.2517	0.1212	0.0939

Dy	proportion	156	158	160	161	162	163	164
Dy-161 spike	0.6296	0.0002	0.0007	0.0023	**0.9575**	0.0251	0.0089	0.0055
Dy-163 spike	0.3704	0.0001	0.0001	0.0003	0.0036	0.0125	**0.9684**	0.0152
Double-spike	0.5625	0.0002	0.0005	0.0016	0.6041	0.0204	0.3643	0.0091
Standard	0.4375	0.0005	0.0009	0.0233	0.1890	0.2549	0.2489	0.2824
Optimal mixture		0.0004	0.0007	0.0111	0.4226	0.1231	0.3139	0.1284

Yb	proportion	168	170	171	172	173	174	176
Yb-166 spike	0.5082	0.0001	0.0038	**0.9507**	0.0261	0.0074	0.0099	0.0021
Yb-167 spike	0.4918	0.0001	0.0003	0.0013	0.0033	0.0078	**0.9840**	0.0036
Double-spike	0.5638	0.0001	0.0021	0.4838	0.0149	0.0076	0.4890	0.0028
Standard	0.4362	0.0012	0.0298	0.1409	0.2168	0.1610	0.3202	0.1299
Optimal mixture		0.0006	0.0142	0.3342	0.1030	0.0745	0.4154	0.0583

a The optimal mixture compositions are obtained through the DS counterspike tests. E-i spike is nominally enriched in ^i^E isotope.

For the counterspike and doping tests of the DS measurements, the solution is measured only once for each condition. The confidence intervals were calculated by propagating the errors of the 40 cycles in each measurement using a Monte Carlo approach. The Monte Carlo error propagation involved (i) generating 500 simulated measured ratios using normal distributions according to the covariance matrix calculated from all the cycles in that measurement, (ii) conducting the DS reduction on the simulated ratios, and (iii) calculating the errors of the results obtained from the reduction.

### 2.5 Sample Selection

Geostandards analyzed in this study include 4 basalts (BCR-2, BHVO-2, BIR-1a, W-2), 1 andesite (AGV-2), 1 granite (G-3), 1 schist (SDC-1), 1 limestone (CCH-1), and 2 manganese nodules (NOD-A-1, NOD-P-1). The sample set also contains an iron formation sample (BIF-311) from Carajás, Brazil.^46^ The geostandards were analyzed under three different conditions over a period of ∼6 years.

(1) BIR-1a, BHVO-2, and G-3 were analyzed for Ce, Nd, Sm, Eu, Gd, Dy, Er, and Yb isotopes using SSB, termed *NonSp* group for nonspike measurements. The basaltic geostandard BCR-2 analyzed in Hu et al.^16^ was measured using the same methodology and evaluated together with samples in the *NonSp* group. Isotopic analyses using SSB require 100% yield to ensure that the isotopes are not fractionated during sample processing. Low yields typically caused by incomplete digestion of the samples or low recovery on columns can induce undesired artificial isotopic fractionation.
(2) AGV-2, BCR-2, BHVO-2, BIR-1a, SDC-1, W-2, CCH-1, NOD-A-1, and NOD-P-1 were analyzed for Ce, Nd, Sm, Gd, Dy, and Yb isotopes using DS, termed *OvSp* group for overlapping spike measurements. The double-spike approach is immune to problems induced by low yields since the isotopes in the spike fractionate with the isotopes in the sample. However, doping multiple spikes especially for REEs adjacent to each other complicates isobaric interference correction. For example, the isobaric interference of ^142^Nd on ^142^Ce can be corrected by monitoring ^144^Nd assuming the introduced Nd impurity has natural abundances. If the sample is doped with Nd spike, the isotopic composition of the Nd impurity in the Ce elution cut will deviate from natural abundances, leading to erroneous isobaric interference correction. For Ce isotopic analysis, we used the isotopic composition of the optimal Nd spike-sample mixture for Nd isobaric interference correction rather than Nd natural abundances, which may not be correct.
(3) AGV-2, BCR-2, and BHVO-2 were analyzed for Ce, Sm, Dy, and Yb isotopes using DS and for Eu and Gd isotopes using SSB. BIF-311 was analyzed for Ce isotopes using DS and for Eu isotopes using SSB (termed *NonOvSp* group for non-overlapping spike measurements). All 4 REEs analyzed using DS do not have any isobaric interference on each other. The isobaric interferences caused by adjacent REEs (e.g., ^142^Nd on ^142^Ce, ^154^Gd on ^154^Sm, and ^164^Er on ^164^Dy) can be corrected using natural abundances.

### 2.6 Spike Addition, Sample Digestion, and Bulk REE Extraction

For the *OvSp* group, spike solutions were added to the samples and dried before digestion. The mass of spike solutions added is based on the sample weights and REE concentrations from the literature. For the *NonOvSp* group, ∼2% aliquots were pipetted after digestion and measured to determine the REE concentrations. The spike solutions were added according to the REE concentrations measured. In either case, the spike-sample mixture is dried to ensure homogenization.

Approximately 50 to 150 mg samples in powder form were digested using a 3:1 mixture of HF and HNO_3_ in closed beakers on a hot plate at 150 °C for a minimum of 48 h. After digestion, the samples were dried at 110 °C, redissolved in aqua regia for 2 h to remove fluorides and organic matter, and then dried again. After a second treatment in aqua regia, the samples were transferred to 3 mol/L HNO_3_ for REE extraction. The digested samples were passed through prepacked TODGA columns using Eichrom’s Vacuum Box System for REE extraction.^3,16^ The yields of REE extraction were near 100%.

### 2.7 Separation of Individual REEs Using the FPLC System

**Figure 1:**
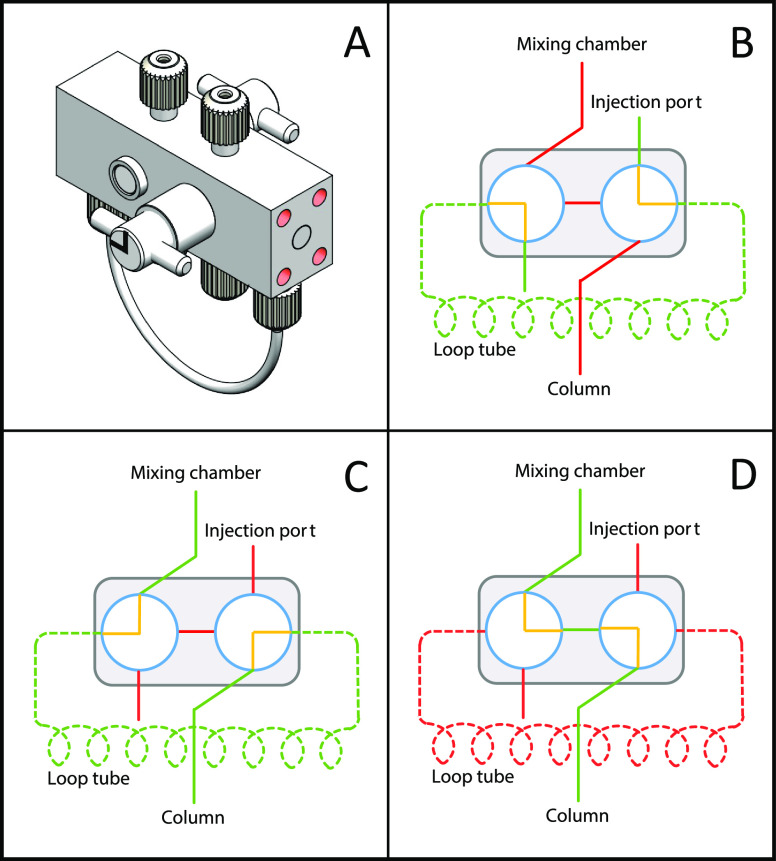
(A) Schematics of the sample loading valve. (B) Sample loading stage: the sample dissolved in 350 to 500 μL of reagent is passed from the injection port and stored in a loop tube. (C) Sample loop filling stage: the sample in the loop tube is pushed into the column with the aid of reagents from the mixing chamber. (D) Elution stage: reagents use a shortcut through the inner path and are directly introduced into the column.

After REE extraction, the separation of REEs from each other was achieved using the FPLC system, which allowed for complete separation of the whole set of REEs.^16,47−49^ The detailed description of the patented FPLC system can be found in Dauphas et al.,^49^ Ireland et al.,^47^ and Hu et al.^16^ A major update of the FPLC system since Hu et al.^16^ is the implementation of a sample loading valve that replaced two 3-way valves originally located between the mixing chamber and column (Figure 1). The sample loading valve is used to alternate between elution and loading states by changing the flow path prior to the column.

The column for REE separation is 70 cm in length and 1.6 mm in inner diameter, filled with 1.4 mL of 25 to 50 μL of Ln-spec resin. The REE elution is conducted at 70 °C with a flow rate of 0.17 mL/min. The chemical purification procedures for *NonSp* and *OvSp* groups are detailed in Hu et al.^16^ REEs after a preliminary FPLC elution were recombined in such a manner that REEs not adjacent to each other were loaded together on a second column (Ce, Nd, Sm+Gd+Er, and Eu+Dy+Yb). This allowed us to run 2 columns for each REE while minimizing the number of procedures (1 + 4 = 5 columns in total compared to 1 + 8 = 9 columns in total if some REEs had not been recombined for a second elution). We ran a third FPLC column for Ce in NOD-A-1 and Nd in NOD-P-1 to further eliminate isobaric interferences.

**Figure 2:**
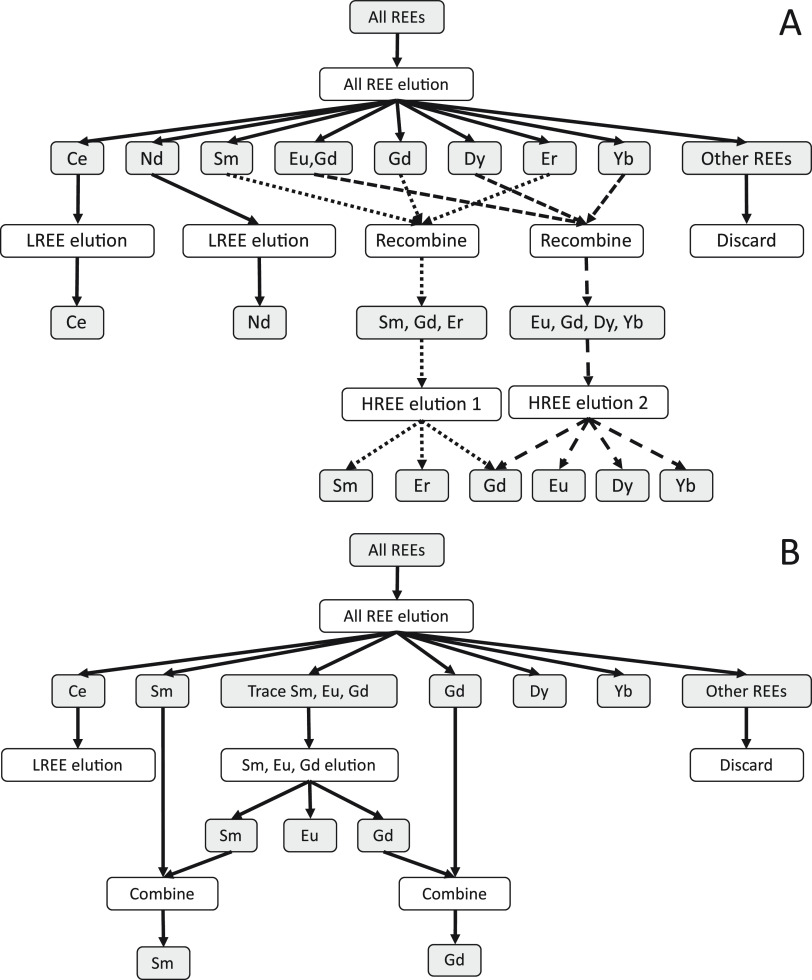
Flowchart of FPLC elution for the *NonSp*, *OvSp*(16) (A) and *NonOvSp* group (B).

**Figure 3:**
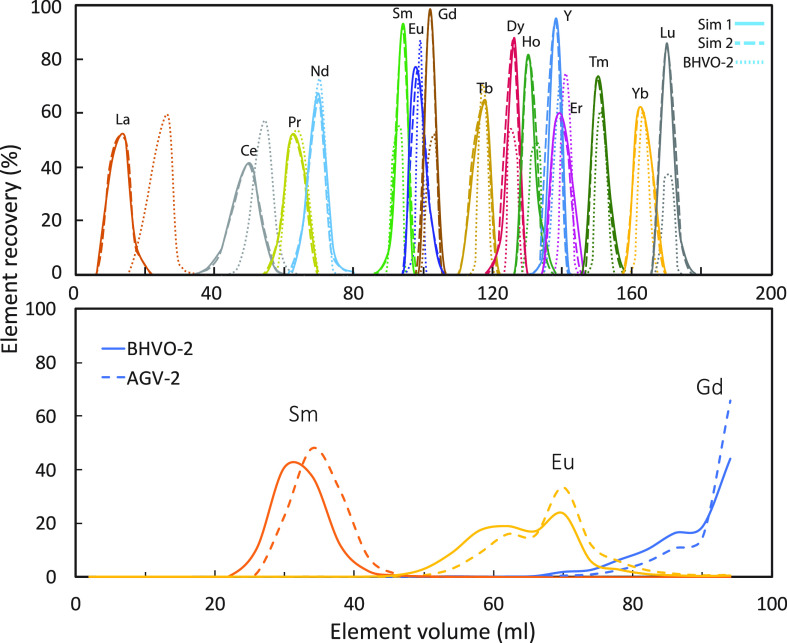
(A) Preliminary FPLC elution curves of the REEs. The acid molarity of the elution solution can be found in Hu et al.^16^ (B) FPLC elution curves for separating Sm, Eu, and Gd for the *NonOvSp* group.

The chemical purification procedures for the *NonOvSp* group are adjusted to further reduce the number of procedures (Figure 2). The extracted REEs were first subjected to a preliminary FPLC elution (Figure 3A). After the first separation, the Ce cut is further purified using a second FPLC elution for Ce separation in Hu et al.^16^ The elution cuts containing all Eu and part of Sm and Gd were combined and subject to a second FPLC elution illustrated in Figure 3B to separate Eu from Sm and Gd, after which the separated Sm and Gd were also recombined with the rest of Sm and Gd separated from the preliminary elution.

The concentrations in all of the elution cuts from the first column were measured to decide which fractions would be recombined for a second pass on the column. To avoid spike contamination, the *NonSp* samples were introduced through two 3-way valves described in Hu et al.,^16^ and the *OvSp* and *NonOvSp* samples were introduced using the sample injection valve (Figure 1). For each group, the resin is reused for all the samples. The yield of each column is over 95% for all REEs.

The overall yield of the full chemical procedure, starting with sample digestion, is 40–70% for most REEs and ∼90% for Eu due to incomplete dissolution of the samples before they are loaded into the FPLC system. Since the partitioning coefficients of REEs on the Ln-Spec resin decrease with increasing HCl molarity, REEs are loaded in very diluted HCl, which can result in incomplete dissolution and potentially isotopic fractionation. After REE extraction, the REEs were dissolved in 1 M HCl, dried to ∼50 μL, and further diluted in 350 to 500 μL of MQ water before loading. The mass loss is not known, but the influence of incomplete dissolution can be evaluated by comparing the DS and SSB measurements.

### 2.8 MC-ICP-MS Analysis

The concentrations and isotopic compositions of REEs were analyzed at The University of Chicago on a Thermo Scientific MC-ICP-MS instrument upgraded to Neptune Plus specifications. The REE concentration measurements were discussed in detail in Pourmand et al.^3^ The cup configurations are listed in Table 2. All REEs except Dy and Yb were measured in static mode. All isotope measurements consisted of a 60 s amplifier baseline measurement and a 60 s take-up time. Analyses of Ce, Nd, Sm, Eu, Gd, and Er isotopic compositions consisted of 41 cycles with an integration time of 8.184 s. The idle time of each cycle was set to 0. The first cycle of each measurement was discarded as the isotopic ratios were unstable with 0 idle time. We used peak jumping to monitor Er interference for Dy isotopes and Er and Hf interferences for Yb isotopes. The subcup configurations used to monitor isobaric interferences were measured in two cycles of 4.142 s integration time at the beginning of each measurement, which were followed by 20 cycles of main cup configuration with 16.368 s integration time. The idle time of cup configuration was set to 10 s wherever the magnetic field and analyzed mass was changed; otherwise, the idle time was set to 0.

**Table 2:** Cup Configurations for the REEs^a^

element		L4	L3	L2	L1	C	H1	H2	H3	H4
Ce	Main	^131^Xe*		^136^Ce	^137^Ba	^138^Ce	^139^La	^140^Ce	^142^Ce	^144^Nd
Nd	Main	^140^Ce*	^142^Nd	^143^Nd	^144^Nd	^145^Nd	^146^Nd	^148^Nd	^150^Nd	^152^Sm*
Sm	Main	^144^Sm	^145^Nd*	^147^Sm	^148^Sm	^149^Sm	^150^Sm	^152^Sm	^154^Sm	^156^Gd*
Eu	Main		^151^Eu	^153^Eu	^156^Dy	^158^Dy	^161^Dy	^162^Dy	^164^Dy	^166^Er*
Gd	Main	^150^Sm*	^152^Gd	^154^Gd	^155^Gd	^156^Gd	^157^Gd	^158^Gd	^160^Gd	^162^Dy*
Dy	Main	^156^Dy*	^157^Gd*	^158^Dy	^160^Dy	^161^Dy	^162^Dy	^163^Dy	^164^Dy	
	Sub	^158^Dy	^159^Tb	^160^Dy	^162^Dy	^163^Dy^n^	^164^Dy	^165^Ho	^166^Er	
Er	Main	^161^Dy*	^162^Er	^163^Dy*	^164^Er	^166^Er	^167^Er	^168^Er	^170^Er	^173^Yb
Yb	Main	^168^Yb*	^170^Yb	^171^Yb	^172^Yb	^173^Yb	^174^Yb	^175^Lu	^176^Yb	^180^Hf*
	Sub	^166^Er	^168^Yb	^169^Tm	^170^Yb	^171^Yb	^172^Yb	^173^Yb^n^	^174^Yb	^178^Hf

a Faraday cups with * are mounted with 10^12^ Ω amplifiers while the ones unlabeled are mounted with 10^11^ Ω amplifiers Faraday cups with superscript n are used to normalize the signals of subconfigurations to the main configurations.

Unless otherwise specified, DS calibration and doping tests were conducted with the purified sample in 0.3 mol/L HNO_3_ + 0.002 mol/L HF introduced into the mass spectrometer by using an Apex Q + Spiro TMD desolvating nebulizer system. We noticed that isotopic analyses using Apex Q + Spiro TMD required an extensive time to wash down the background (∼10 min rinsing time for a sample). We later switched to a CETAC Aridus I desolvating nebulizer system for isotopic analyses of the *OvSp* group and managed to reduce the rinsing time to 4 min. The Aridus I was later upgraded to an Apex Omega desolvating nebulizer system and used for isotopic analyses of the samples from the *NonSp* and *NonOvSp* groups. The *NonSp* group was analyzed before the *NonOvSp* group, so that the introduction system, nebulizer, and cones were all free of spikes. Overall, the sensitivities using Apex Omega and Aridus I are comparable and are both better than Apex Q + Spiro TMD. However, no systematic difference in the quality of the isotopic analyses was observed with the three desolvating nebulizer systems.

Data reduction for SSB was done by copying the raw data into a spreadsheet for correction of blanks and isobaric interferences. Data reduction for DS was done using a Mathematica code, which can automatically read the raw intensities from an input file (CSV, xls, xlsx) and conduct blank correction, isobaric interference correction, DS reduction through exact solving of an equation set of 4 isotopes, DS minimization using 4 or more isotopes, and error propagation using a Monte Carlo procedure.

In the *OvSp* group, some of the REEs that cause isobaric interference were spiked. As mentioned in Section 2.5, if an element causing isobaric interference is spiked, then the isotope abundance of that element is assumed to be that of its optimal spike-sample mixture. For example, to correct ^142^Nd for ^142^Ce, ^145^Nd was monitored and used to subtract ^142^Nd assuming the isotope composition of the Nd impurity is the isotope composition of the optimal spike-sample mixture rather than natural abundances. The influence of the correction can be evaluated by comparing *OvSp* and *NonOvSp* measurements.

### 2.9 Correction for the Double-Spike Approach

In the conventional DS reduction, all four isotopes are integrated into a set of 3 equations to obtain the natural isotopic fractionation. Ideally, elements that can form isobaric interference are eliminated during the sample processing stage. Isotopes of adjacent elements that induce isobaric interferences are also routinely monitored to subtract the isobaric interferences based on their respective isotope abundances. In practice, however, a trace amount of isobaric interference may persist in the purified sample. Subtraction of isobaric interferences by monitoring isotopic masses other than those of the element of interest is not always feasible or reliable, in part due to the low isotopic abundance of some of those monitored interferences, which propagates into a large uncertainty on the correction. For example, the correction of ^138^Ba (∼71.7% in abundance) on ^138^Ce by monitoring ^137^Ba (∼11.23%) is challenging because any uncertainty or inaccuracy in the ion intensity measured at mass 137 is magnified by a factor of ∼7 when calculating the intensity of ^138^Ba^+^.

**Figure 4:**
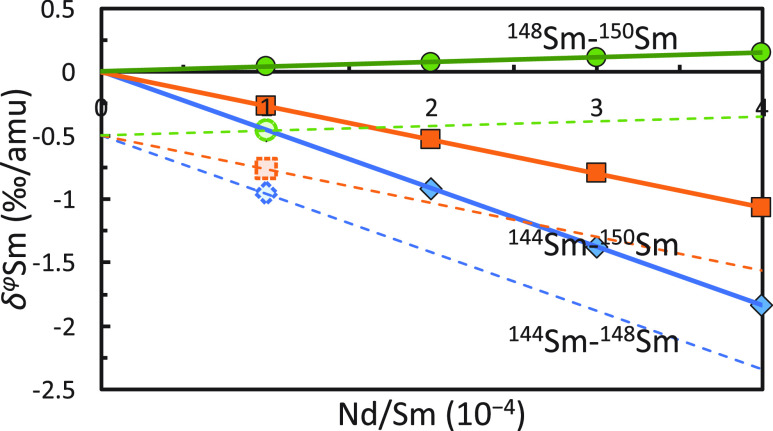
Simulated spurious apparent isotopic fractionations as a function of isobaric interference for Sm DS reduction by using different combinations of isotopes. The circle, cube, and diamond points are apparent isotopic fractionations calculated by DS reduction using ^147^Sm–^148^Sm–^149^Sm–^150^Sm, ^144^Sm–^147^Sm–^149^Sm–^150^Sm, and ^144^Sm–^147^Sm–^148^Sm–^149^Sm after the intensities of a spike-standard measurement were adjusted to simulate the effect of Nd isobaric interference. The apparent isotopic fractionations calculated by DS reduction using different isotopes are all linear to the Nd/Sm molar ratio and cross the same point (true natural isotopic fractionation). The standard used for simulation in this figure has no isotopic fractionation (crossing point of the solid lines). If the sample has positive or negative true isotopic fractionation, the apparent isotopic fractionation of all 3 versions of DS reductions (dashed symbols) will fall on dashed lines that are parallel to the corresponding solid lines.

For isotopic systems with more than 4 measurable isotopes, additional isotopes provide extra constraint to correct for isobaric interferences (see also ref (45)). If the isotopic composition of the interfering species is known, it becomes possible to use the additional ratios analyzed (beyond the minimum of 3 ratios necessary for DS reduction) to account for the contribution of the interference. By measurement of a greater number of isotopic ratios, additional equations are added to the system, thus allowing for the resolution of more unknowns. A limitation of this method is that the exact nature of the interference may not always be known beforehand. In the section below, we introduce an alternative method for addressing such scenarios. This approach involves working with an expanded system in which three subsystems are solved. Each subsystem is formed by combining three ratios under the assumption that all isobaric interferences have been corrected. If an interference is indeed present, then the three subsystems will produce distinct isotopic fractionations. The patterns observed in these fractionations can offer insight into the nature of the interference. Taking samarium as an example, ^144^Sm, ^147^Sm, ^148^Sm, ^149^Sm, and ^150^Sm can be used for DS reduction. We can form 3 sets of 4 isotopes ^144^Sm–^147^Sm–^149^Sm–^150^Sm, ^147^Sm–^148^Sm–^149^Sm–^150^Sm, and ^144^Sm–^147^Sm–^148^Sm–^149^Sm that contain the spiked isotopes ^147^Sm and ^149^Sm and can be used for solving exactly the DS equations. If no interference is present, then these subsystems should yield identical natural isotopic fractionation. However, contamination with Nd would create isobaric interferences on ^144^Sm, ^148^Sm, and ^150^Sm from ^144^Nd, ^148^Nd, and ^150^Nd, giving rise to spurious isotopic fractionations for all three sets of DS reductions. Solving for a system of 4 equations with 4 unknowns (including the contribution of Nd on Sm) would yield a solution. As discussed below, the virtue of using the alternative approach of solving 3 systems for 3 unknowns is that we can test different hypotheses for the nature of potential isobaric interferences (Figure 4)

We start by establishing a relationship between the apparent isotopic fractionation and the intensity of the isobaric interference for DS reduction using a certain combination of isotopes. Spurious apparent isotopic fractionation can be caused by isotopic anomalies and isobaric interferences
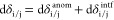
7where dδ_i/j_^anom^ is the spurious fractionation of isotopic ratio ^i^E/^j^E induced by nucleosynthetic anomalies (eqs 30–33 in Hu and Dauphas^39^) and dδ_i/j_^intf^ is the spurious isotopic fractionation induced by isobaric interferences, which takes the form
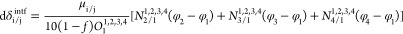
8where
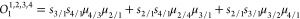
9
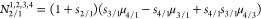
10
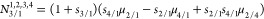
11
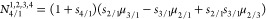
12with , and isobaric interferences φ_i_ = 10^4^ d*I*_i_/*I*_i_ written as the ratio of intensity variation d*I*_i_ and intensity *I*_i_ in the measurement (details in associated text). One application of eq 8 is to evaluate how vulnerable the combination of isotopes for DS reduction is to isobaric interference in order to choose the most robust combination of isotopes.

Below, we will derive a relationship of true natural isotopic fractionation and the apparent isotopic fractionations using different combinations of isotopes for DS reduction. Assuming that the isotopic abundance of Nd contamination is known, with intensities of interferences being φ_144_, φ_146_, and φ_150_ on ^144^Sm, ^148^Sm, and ^150^Sm for molar ratio Nd/Sm = η_0_, the intensity of interferences are then φ_144_η/η_0_, φ_146_η/η_0_, and φ_150_η/η_0_ for molar ratio Nd/Sm = η.

According to eq 8, the spurious isotope fractionation induced by isobaric interferences for ^144^Sm–^147^Sm–^149^Sm–^150^Sm can be written as,
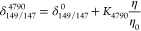
13where δ_149/147_^0^ is the isotope fractionation free of isobaric interference from Nd and *K*_4790_ is a constant,
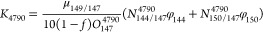
14

Equations 13 and 14 show that the apparent isotope fractionation scales linearly with the interference level (Nd/Sm here; Figure 4). Similarly, DS reductions using ^147^Sm–^148^Sm–^149^Sm–^150^Sm and ^144^Sm–^147^Sm–^148^Sm–^149^Sm follow, respectively,
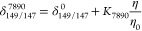
15
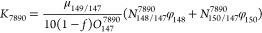
16
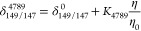
17
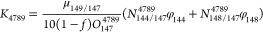
18

Combining eqs 13, 15, and 17 gives
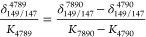
19

Combining any two equations out of eqs 13, 15, and 17 gives the true isotope fractionation. For eqs 14 and 16 as an example
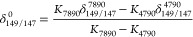
20

By solving the 3 subsystems of 3 equations assuming no interference, we can test hypotheses for the nature of possible interferences by comparing how the solutions differ (provided that the interference is large enough). Having established that, we can then solve the full system of 4 equations with 4 unknowns to correct for the presence of isobaric interferences.

We determined the candidate of potential isobaric interference for REEs based on the doping tests. We then applied the same approach to all elements and the analysis identified the following interferences for Nd, Sm, Gd, and Yb isotopes in the *OvSp* group: (i) ^144^Sm, ^148^Sm, and ^150^Sm on ^144^Nd, ^148^Nd, and ^150^Nd, (ii) ^144^Nd, ^148^Nd, and ^150^Nd on ^144^Sm, ^148^Sm, and ^150^Sm, (iii) ^140^Ce^16^O and ^142^Ce^16^O on ^156^Gd and ^158^Gd, and (iv) ^155^Gd^16^O, ^156^Gd^16^O, ^157^Gd^16^O, ^158^Gd^16^O, and ^160^Gd^16^O on ^171^Yb, ^172^Yb, ^173^Yb, ^174^Yb, and ^176^Yb. Note that for Nd and Sm isotope measurements, Sm and Nd intensities are monitored and corrected according to the isotope compositions of the spike-sample mixture. As stated previously, this way, Sm and Nd impurities with natural abundances are not considered in the Nd and Sm cuts. We hereby use natural abundances for the Sm and Nd interferences in the DSCII to adjust deviations from the spike-sample mixture. For Gd and Yb, the isobaric interference corrections for Ce and Gd oxides are based on the isotope compositions of Ce and Gd spike-sample mixtures. These were corrected for in the isotopic analyses, and the results are reported in Table 3.

**Table 3:** REE Isotope Fractionations of Analyzed Geostandards

sample	Ce	Nd	Sm	Eu	Gd	Dy	Er	Yb
Not Spiked (*NonSp*)								
BCR-2	0.024 ± 0.015	–0.009 ± 0.023	0.004 ± 0.007	0.015 ± 0.063	0.019 ± 0.026	0.018 ± 0.026	–0.050 ± −0.021	0.022 ± 0.050
BIR-la	0.055 ± 0.006	–0.217 ± 0.035	–0.040 ± 0.040	–0.026 ± 0.029	–0.002 ± 0.036	0.027 ± 0.008	–0.101 ± −0.054	0.036 ± 0.025
BHVO-2	0.003 ± 0.011	–0.068 ± 0.016	–0.015 ± 0.025	0.027 ± 0.010	0.007 ± 0.010	0.042 ± 0.007	–0.094 ± −0.011	–0.041 ± 0.118
G-3	0.027 ± 0.007	–0.122 ± 0.017	–0.089 ± 0.047	–0.123 ± 0.021	0.006 ± 0.011	0.040 ± 0.021	–0.078 ± −0.051	0.077 ± 0.037
Ce, Nd, Sm, Gd, Dy, and Yb Spiked (*OvSp*)								
AGV-2	0.035 ± 0.011	0.014 ± 0.020	–0.052 ± 0.018		0.013 ± 0.008	–0.020 ± 0.007		–0.009 ± 0.010
BCR-2	0.024r ± 0.010	–0.001 ± 0.010	–0.015 ± 0.015		0.018 ± 0.004	–0.026 ± 0.010		–0.020 ± 0.023
BHVO-2	0.035 ± 0.018	–0.004 ± 0.022	–0.008 ± 0.025		0.028 ± 0.007	–0.012 ± 0.005		0.001 ± 0.018
BIR-la	–0.044 ± 0.012	0.023 ± 0.015	0.049 ± 0.021		0.036 ± 0.005	0.003 ± 0.007		0.005 ± 0.007
SDC-1	0.008 ± 0.016	–0.009 ± 0.016	–0.037 ± 0.022		0.011 ± 0.005	–0.015 ± 0.003		–0.025 ± 0.014
W-2	–0.005 ± 0.012	0.009 ± 0.009	–0.025 ± 0.011		0.019 ± 0.005	–0.023 ± 0.006		0.002 ± 0.006
CCH-1	0.028 ± 0.008	0.071 ± 0.013	0.011 ± 0.008		0.043 ± 0.010	0.032 ± 0.004		0.050 ± 0.006
CCH-1^a^								0.041 ± 0.006
NOD-A-1		0.063 ± 0.011	0.037 ± 0.006		0.031 ± 0.006	0.004 ± 0.004		0.007 ± 0.008
NOD-A-l^b^	0.085 ± 0.010		0.004 ± 0.009		0.022 ± 0.013	0.003 ± 0.007		0.003 ± 0.003
NOD-P-1			0.018 ± 0.008		–0.017 ± 0.006	0.005 ± 0.004		0.005 ± 0.005
NOD-P-1^a^		0.031 ± 0.019	0.027 ± 0.006					
Ce, Sm, Dy, and Yb Spiked (*NonOvSp*)								
AGV-2	0.037 ± 0.007		–0.008 ± 0.005	0.024 ± 0.034	0.023 ± 0.005	0.001 ± 0.006		–0.018 ± 0.004
BCR-2	0.006 ± 0.018		–0.004 ± 0.006	0.060 ± 0.034	0.016 ± 0.012	0.009 ± 0.004		–0.035 ± 0.006
BHVO-2	0.048 ± 0.004		0.012 ± 0.004	0.026 ± 0.034	0.024 ± 0.008	0.012 ± 0.002		–0.004 ± 0.006
BIF-311	0.032 ± 0.017		0.153 ± 0.034					

a Samples that are purified one more time.

b Duplicated samples or measurements.

### 2.10 Choices of Isotopes for Double-Spike Data Reduction and Correction

To use the DS approach for data reduction, at least 4 isotopes are required, with two of them being enriched isotopes in the spike solution. Using 4 isotopes, a set of 3 versions of eq 5 can be solved to determine the isotopic ratios of the samples. Isotopes used for the DS reduction need to be abundant enough in the spike-sample mixture to be measured accurately and not to vary for other reasons such as radiogenic effects and mass-independent fractionation if the isotopic composition of nonspiked samples is not analyzed. Ideally, the isotopes should also be immune to isobaric interferences.

Besides spiked isotopes, if we only consider nonradiogenic REE isotopes with more than 1% in abundance in the spike-sample mixture, candidates include ^140^Ce and ^142^Ce for Ce, ^144^Nd, ^148^Nd, and ^150^Nd for Nd, ^144^Sm, ^148^Sm, ^150^Sm, ^152^Sm, and ^154^Sm for Sm, ^154^Gd, ^156^Gd, ^158^Gd, and ^160^Gd for Gd, ^160^Dy, ^162^Dy, and ^164^Dy for Dy, and ^170^Yb, ^172^Yb, ^173^Yb, and ^176^Yb for Yb.

**Figure 5:**
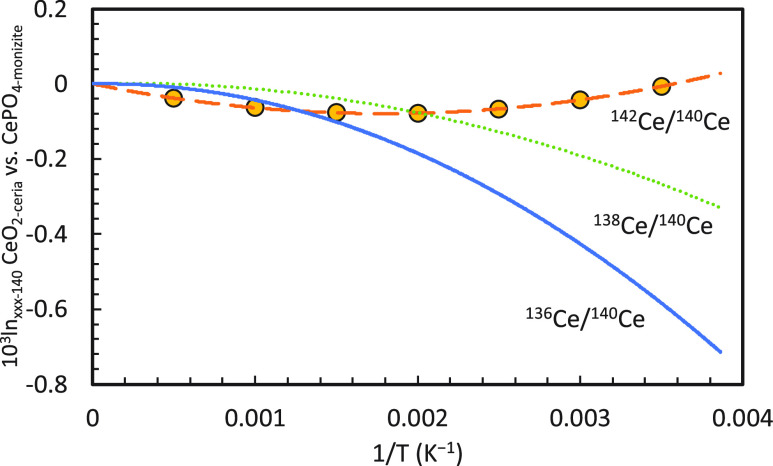
Influence of ^136^Ce/^140^Ce and ^138^Ce/^140^Ce MDF on the DS reduction of ^142^Ce/^140^Ce. This figure is modified from Figure 3 in Schauble.^26^ The solid, short-dashed, and long-dashed lines are, respectively, equilibrium isotopic fractionations between Ce^4+^-bearing CeO_2_-ceria (Ce-cerianite) and Ce^3+^-bearing CePO_4_-monazite for ^136^Ce/^140^Ce, ^138^Ce/^140^Ce, and ^142^Ce/^140^Ce, calculated as the sum of the nuclear volume and mass-dependent components. The orange points are the simulated natural isotopic fractionations if one passes the Ce isotopic composition in the figure to a DS reduction, which are equal to the total isotopic fractionation of ^142^Ce/^140^Ce. Adapted with permission from ref (26). Copyright 2023 The Geochemical Society of Japan.

Cerium has 4 isotopes, ^136^Ce, ^138^Ce, ^140^Ce, and ^142^Ce, among which ^138^Ce is a radiogenic daughter of ^138^La. The range of ε^138^Ce in the terrestrial samples is ∼2 epsilon (0.2‰).^50^ Schauble^26^ pointed out that ^142^Ce has a nuclear charge radius that stands out relative to ^136^Ce, ^138^Ce, and ^140^Ce, giving rise to anomalous (nonmass dependent) isotopic fractionation of ^142^Ce/^140^Ce compared to ^136^Ce/^140^Ce and ^138^Ce/^140^Ce. The majority of ^136^Ce (98.97%) and ^138^Ce (97.97%) comes from the spike in the optimal spike-sample mixture. As a result, the influence of naturally occurring ^136^Ce and ^138^Ce on the DS reduction is negligible for terrestrial samples (Figure 5). As the spiked isotopes dominated the ^136^Ce and ^138^Ce contribution in the spike-sample mixture, what the DS reduction actually measured is the isotope ratio of ^142/140^Ce.

For Sm, ^144^Sm, ^148^Sm, and ^150^Sm are sensitive to isobaric interferences from Nd isotopes in the DS reduction because (i) the abundances of Nd in rocks are often an order of magnitude higher than those of Sm and (ii) column chemistry tends to leave trace amounts of Nd in the Sm cut since Sm is eluted after Nd. For the *OvSp* group, the problem of Nd interference is further complicated as the isotope abundance of Nd impurity in the Sm cut is not well constrained. Isobaric interferences of ^152^Gd and ^154^Gd on ^152^Sm and ^154^Sm are relatively minor based on the doping tests. We confirmed that Nd caused isobaric interferences. In both *OvSp* and *NonOvSp* groups, ^144^Sm–^147^Sm–^148^Sm–^149^Sm–^150^Sm are used for DS reduction with DSCII. Similarly, ^144^Nd–^145^Nd–^146^Nd–^148^Nd–^150^Nd are used for DS reduction with DSCII in the *OvSp* and *NonOvSp* groups.

For Gd, ^154^Gd is not used in the DS reduction due to the relatively low abundance (∼1.02%) and isobaric interference from ^154^Sm, leaving ^156^Gd, ^158^Gd, and ^160^Gd for the DS reduction. Our doping tests have shown that Gd is most sensitive to isobaric interferences from Ce oxide. We used DSCII to identify that CeO^+^ indeed caused isobaric interference in the Gd DS reduction in the *OvSp* group. Therefore, the DS reduction using ^155^Gd–^156^Gd–^157^Gd–^158^Gd–^160^Gd is corrected for Ce oxides with Ce isotope composition of a spike-sample mixture. In the *NonOvSp* group, ^155^Gd–^157^Gd–^158^Gd–^160^Gd is used for regular DS reduction as no evidence for any interference was found.

For Dy, ^160^Dy is discarded due to the low abundance (∼1.11%) and isobaric interference from ^160^Gd so ^162^Dy and ^164^Dy are used for the DS reduction. Both the *OvSp* and *NonOvSp* groups are analyzed using the regular DS reduction using ^161^Dy, ^162^Dy, ^163^Dy, and ^164^Dy.

For Yb, ^170^Yb is not used due to its low abundance and isobaric interference from ^170^Er, leaving ^172^Yb, ^173^Yb, and ^176^Yb for the DS reduction. Doping tests show that Yb isotopes are most sensitive to GdO^+^, which is confirmed through DSCII. In the *OvSp* group, ^171^Yb–^172^Yb–^173^Yb–^174^Yb–^176^Yb is used for DS reduction to correct Gd oxides with a Gd isotope composition of a spike-sample mixture. In the *NonOvSp* group, a similar DS reduction setting is applied to correct Gd oxide with Gd natural isotope abundances.

## 3 Results

### 3.1 Double-Spike Calibration and Doping Tests

**Figure 6:**
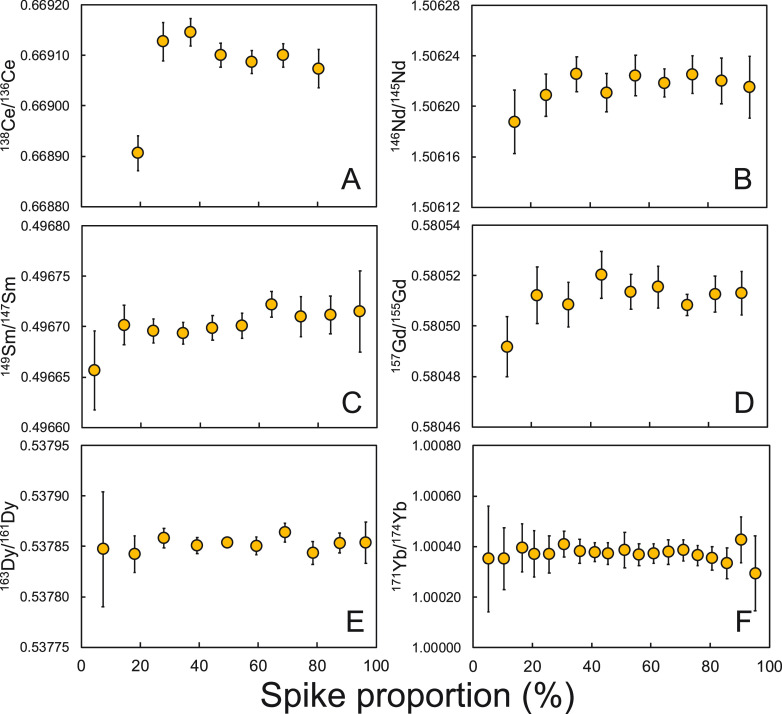
Calibration of double-spike compositions for the REEs. The *y*-axis is the isotope ratio of the two enriched isotopes in the spikes. The *x*-axis is the proportion of the spike in the spike-standard mixture.

**Figure 7:**
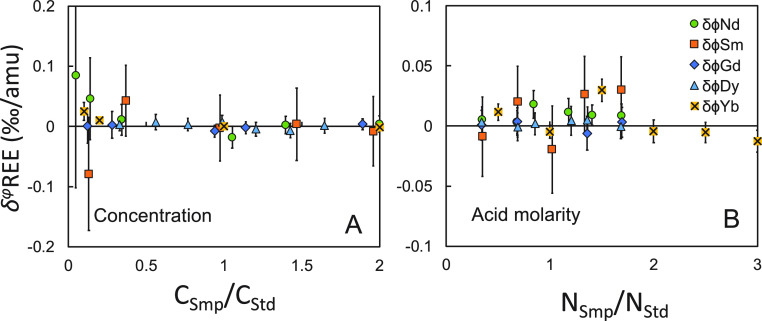
Concentration and acid molarity match tests. Isotopes used for the DS reduction can be found in Table 1. The isotope ratios of the spike-sample mixtures are bracketed by those of spike-standard mixtures.

**Figure 8:**
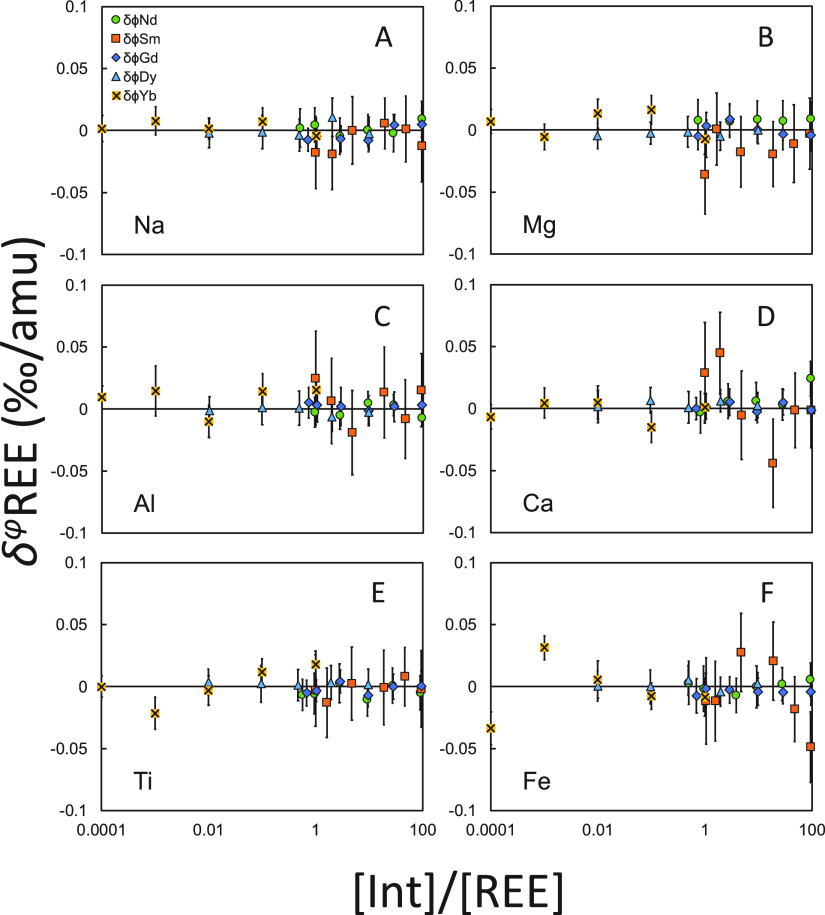
Matrix doping tests (Na, Mg, Al, Ca, Ti, and Fe) are for REEs. Isotopes used for the DS reduction can be found in Table 1. The isotope ratios of the spike-sample mixtures are bracketed by those of spike-standard mixtures.

**Figure 9:**
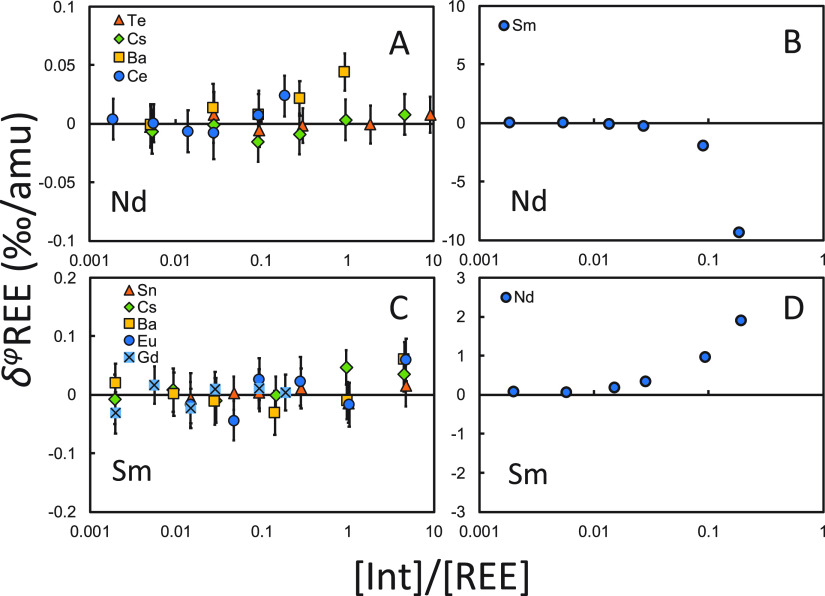
Interference doping tests for Nd and Sm. Isotopes used for the DS reduction can be found in Table 1. The isotope ratios of the spike-sample mixtures are bracketed by those of spike-standard mixtures.

**Figure 10:**
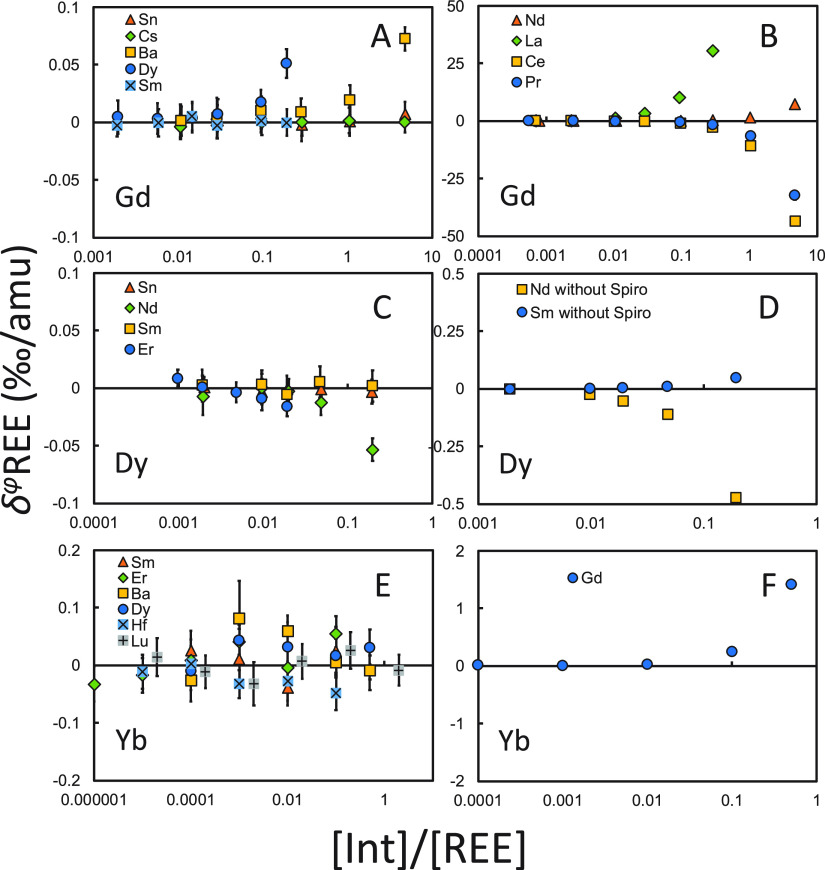
Interference doping tests for Gd, Dy, and Yb. Isotopes used for the DS reduction can be found in Table 1. The isotope ratios of the spike-sample mixtures are bracketed by those of the spike-standard mixtures.

The results of DS calibration and doping tests are presented in Figures 6–10. For the DS calibration, the standard and spike were mixed and analyzed in proportions ranging from an ∼10% spike to a 90% spike (Figure 6). The DS mixtures show large tolerance of mixing ratios ranging from ∼40 to 80% for the REEs, with the optimal spike proportion varying from 60% to 75%.

Isotope analyses using SSB typically require that the concentrations and acid molarities of the samples be close to those of the bracketing standards.^51^ However, our tests show that isotopic analyses using DS are not sensitive to a mismatched concentration and acid molarity (Figure 7).

The concentration tests showed that DS yields accurate results if the concentrations of the samples were between 40% and 200% of those of the standards. Samples dissolved in 0.1 to 0.5 mol/L HNO_3_ show consistent results compared to the standard in 0.3 mol/L HNO_3_ for all the REEs.

Doping tests on major elements, including Na, Mg, Al, Ca, Ti, and Fe, showed that DS was not sensitive to matrix effects (Figure 8). No observable effects have been found for samples doped with up to 100 times REE concentrations for Na, Mg, Al, Ca, Ti, and Fe.

Doping tests show that REE isotope analyses are not sensitive to isobaric interference from nitrides and argides (Figures 9 and 10). Inaccuracy of isotopic measurements comes mostly from the impurity of adjacent REEs, as direct isobaric interferences (e.g., Nd on Ce, Nd on Sm) and isobaric interferences in the form of oxides (e.g., LaO, PrO, CeO, and NdO on Gd; GdO on Yb).

### 3.2 Geostandard Measurements

**Figure 11:**
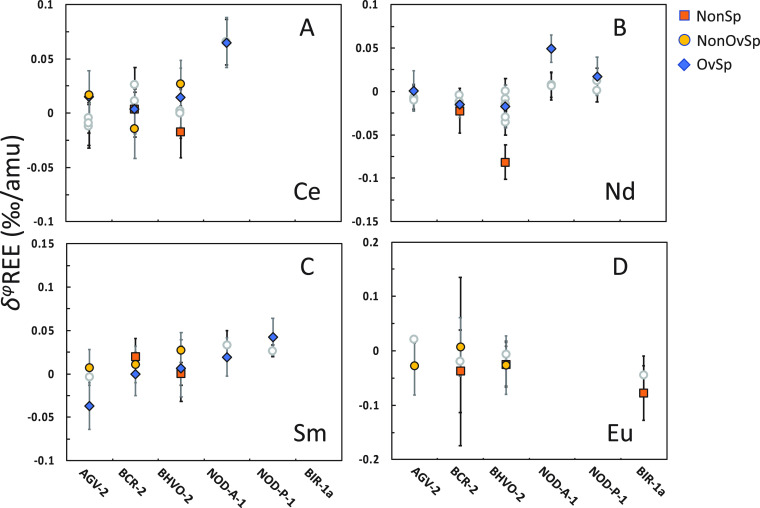
Ce, Nd, Sm, and Eu isotope fractionations analyzed in this study compared to literature values relative to OL-REE. Ce: refs (52−54); Nd: refs (25, 31, 52, and 55−59); Sm: ref (34); Eu: refs (35 and 36).

The stable isotopic compositions of the geostandards measured in this study are listed in Table 3. We compared our measurements with the reported Ce, Nd, Sm, and Eu isotope data in Figure 11. Since the reference materials used in the literature and the OL-REEs were different, we normalized published isotopic data to OL-REEs using BCR-2 and BHVO-2 that were measured here and in previous studies. All the analyzed Ce and Nd isotopes were subtracted by the difference of BCR-2 in the *OvSp* group and BCR-2 analyzed in Bai et al.^52^ Samarium isotopes were subtracted by the difference of BCR-2 in the *OvSp* group and Bai et al.^34^ Due to the relatively low precision of the reported BCR-2 value, Eu stable isotopes were subtracted by the difference of BHVO-2 in the *NonSp* group and Lee and Tanaka.^35^ Our measurements in this study are overall consistent with previously reported values except for Nd isotopes in NOD-A-1 with a difference of ∼0.05‰/amu.

## 4 Discussion

### 4.1 Comparing *NonSp*, *OvSp*, and *NonOvSp* Measurements

**Figure 12:**
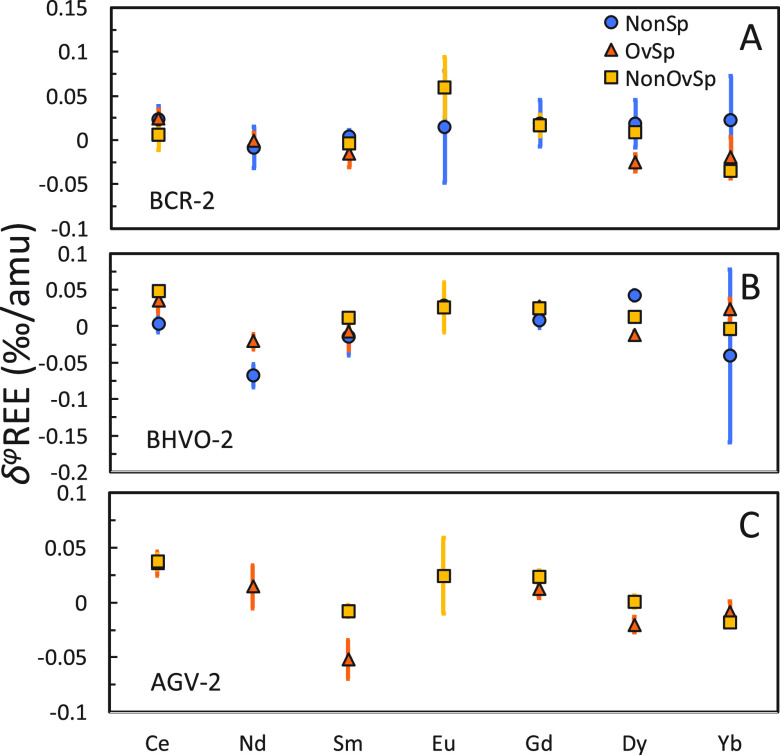
Comparison of REE isotope fractionations of BCR-2, BHVO-2, and AGV-2 from *NonSp*, *OvSp*, and *NonOvSp* groups.

In this section, we assess the accuracy of isotope measurements using multiple spikes by comparing *NonSp*, *OvSp*, and *NonOvSp* measurements (Figure 12). The *NonSp* group is free of spikes and can be compared to the *OvSp* and *NonOvSp* groups to evaluate the influence of isotope fractionation induced by sample digestion, column chemistry, and instrumental measurements. The *NonOvSp* group includes only REEs that are not adjacent to each other, making the correction of isobaric interferences relatively straightforward. The *OvSp* group can be compared to the *NonOvSp* group to assess the influence of isobaric interferences in the presence of multiple spikes. To achieve this goal, we compared REE measurements of BCR-2 and BHVO-2, which are analyzed in all three groups, and AGV-2, which are analyzed in both *OvSp* and *NonOvSp* groups.

For the Ce isotopes, all three groups yield highly consistent results within CIs for BCR-2, BHVO-2, and AGV-2. However, the Nd isotope measurement in the *NonSp* group gives more negative values compared to that in the *OvSp* group for BHVO-2. Fractionated Nd isotopes have previously been observed in the duplicates with very low yields in Hu et al.^16^ Given that the yields on the column are over 95% for Nd in this study, the fractionation most likely took place during incomplete dissolution before loading to the FPLC system, since the media for loading the REEs is very dilute. This incomplete dissolution problem can potentially be addressed using the TODGA resin to separate REEs instead. Since the distribution coefficients of REEs on the TODGA are over 100 for all REE in 3 M HCl,^60^ one can use 3 M HCl as the loading media to ensure complete digestion before loading (e.g., ref (54)). For Sm isotopes, all three groups yield consistent results except for AGV-2 in *OvSp* and *NonOvSp* groups with a difference of ∼0.04‰/amu. This is due to the Nd isobaric interferences in the AGV-2 *NonOvSp* measurement (0.00145 for ^145^Nd/^147^Sm) after using the new chemical procedure.

Overall, all three groups show relatively consistent results for most REEs analyzed. Neodymium and potentially Ce isotopes analyzed using SSB can be affected by the low yield during sample processing and can benefit from the DS approach for accurate measurements. In terms of precision, SSB shows comparable or slightly larger errors than DS for LREEs, while the errors of SSB are much larger than those of DS for HREEs, primarily because the sample amount is more limited due to the substantially lower concentrations of HREEs in nature. This is also the case for Eu isotopes. Europium only has two isotopes and can only be analyzed using SSB. The limited sample amount led to larger errors for Eu isotope analyses compared to other REEs. In the case that a good yield and stable instrumental status is achievable, SSB can provide a precise result like DS. If the sample amount is more limited, the higher precision DS provided can be critical for sample analyses as REE natural isotope fractionations are generally small. However, one should be cautious about potential isobaric interferences that can cause high-precision erroneous results for DS.

### 4.2 REE Isotope Fractionation in Nature

There has been a growing interest in stable isotope analyses of rare earth elements (REEs), including Ce,^16,18,28,29,52−54,61−63^ Nd,^16,25,30−32,52,55,57−59,64−66^ Sm,^16,33,34^ Eu,^16,26,35−37^ Gd,^16^ Dy,^16^ Er,^16,38^ and Yb.^16,38^

Europium displays notable variations in isotope fractionation during magmatic processes, ranging from about −0.150‰/amu to 0.100‰/amu.^27^ As revealed by NRIXS measurements on Mössbauer isotopes ^151^Eu and ^161^Dy in Hu et al.,^17^ equilibrium MDF of Eu and Dy at magmatic temperatures is likely to be negligible. Extrapolation based on lanthanide ionic radii also suggests limited isotope fractionations for other REEs in magmatic processes. However, Schauble^26^ predicted that equilibrium isotope fractionation induced by NFS dominates over equilibrium MDF for Eu.^67,68^ As the isotopic fractionation induced by NFS occurs in the redox reaction and goes in the opposite direction of equilibrium MDF, light isotopes tend to be enriched in Eu^3+^ compared to Eu^2+^. During its crystallization, plagioclase tends to be enriched in Eu^2+^ and heavy isotopes, while the parental melt is depleted in Eu^2+^ and enriched in light isotopes, as is observed (Figure 3 in ref (27)). This is also consistent with the heavy Eu isotopes observed in the iron formation sample BIF-311, which exhibits a mixture of seawater and a hydrothermal REE pattern. The positive Eu anomaly of BIF-311 is from the Eu^2+^ enriched and potentially light Eu isotopes of hydrothermal fluids. Since NFS scales with 1/*T* in K, measurable equilibrium isotope fractionation induced by NFS can persist at relatively high temperature (∼0.1‰/amu between Eu^2+^ and Eu ^3+^ at 1500 K^26^).

Nuclear field shift also causes the subdued isotope fractionation of ^142/140^Ce compared to ^136/140^Ce and ^138/140^Ce at low temperature since more eminent effects of NFS partially canceled equilibrium MDF on ^142/140^Ce.^26^ As mentioned in Section 2.10, the isotopic fractionation reported using ^136^Ce and ^138^Ce DS is calculated from ^142/140^Ce (also see ref (63)), while all the isotope analyses using SSB also only reported ^142/140^Ce due to the extremely low natural abundances of ^136^Ce and ^138^Ce.^16,52−54^ In the discussion below, we consider only Ce isotope fractionation reported as ^142^Ce/^140^Ce. As the NFS scales with 1/*T* and equilibrium MDF scales with 1/*T*^2^, the maximum equilibrium isotope fractionation of ^142/140^Ce is achieved at medium temperature (∼550 K; Figure 3 in ref (26)). In terms of natural samples, Ce shows stable isotope fractionation with a range over 0.3‰/amu in ferromanganese oxy/hydroxide.^29,53^ As soluble Ce^3+^ is oxidized to Ce^4+^ and adsorbed onto manganese oxide/hydroxide (potentially iron oxide/hydroxide), ^142^Ce is expected to be enriched in Ce^4+^ relative to ^140^Ce, which is consistent with the adsorption experiments.^28^ However, currently reported ferromanganese oxy/hydroxides show higher ^142^Ce/^140^Ce compared to igneous rocks.^29,53^ In this study, we also found slightly heavier Ce isotopes in the ferromanganese deposit NOD-A-1 (0.085‰/amu) relative to those in igneous rocks. One dolomite sample (JDo-1) shows resolvable Ce (0.061‰/amu) isotope fractionation,^52,53^ while carbonate CCH-1 analyzed in this study shows negligible Ce (0.028‰/amu) isotopic fractionation. The enrichment of ^142^Ce in ferromanganese oxy/hydroxides were explained as a reservoir effect in Nakada et al.:^29^ as seawater ^142^Ce is progressively enriched during adsorption near the seaside, the ferromanganese oxy/hydroxide inherits the ^142^Ce enriched signature in the deep ocean. The role of kinetic isotope fractionation, however, also needs to be examined. The iron formation BIF-311 shows negligible Ce isotope fractionation (0.032‰/amu).

Published data on Nd isotopes in the igneous rocks show a range from −0.062‰/amu to 0.081‰/amu, with most data centered within 0.05‰/amu. The two most fractionated Nd measurements (−0.062‰/amu^25^ and 0.081‰/amu^30^) are both attributed to kinetic isotope fractionation through boundary diffusion^30^ and interaction with reactive porous flow.^25^ Neodymium (and Sm) isotopes are also expected to fractionate during the low-temperature adsorption process.^18^ Dolomite sample JDo-1 displays slightly Nd heavy isotope composition (∼0.064‰/amu^52,53^). The carbonate CCH-1 analyzed in this study also shows heavy Nd isotopes (0.071‰/amu). Bai et al.^32^ also reported fractionated Nd isotopes in a basaltic soil profile on Hainan Island, South China, spanning ∼0.105‰/amu. Overall, Nd isotope fractionation is relatively limited and occurs mostly in low-temperature processes such as adsorption/precipitation and chemical weathering or controlled by kinetic processes.

The ranges of Er and Yb isotope fractionation of published data are 0.025‰/amu to 0.125‰/amu and −0.05‰/amu to 0.15‰/amu,^38^ respectively, with Yb fractionation attributed to garnet crystallization. Sm, Gd, and Dy show negligible isotope fractionation due to limited measurements.

Overall, REE isotopes tend to fractionate in low-temperature environments, while magmatic processes induce limited isotope fractionation for REEs except Eu. Nonetheless, measurable REE stable isotope fractionation may still be preserved at high temperature due to kinetic processes.

## 5 Conclusions

In this contribution, we present a set of new methods to analyze the stable isotope fractionations of 8 REEs. All of the REEs are separated from each other using the FPLC system. The isotopic fractionations reported in this work indicate that REE isotopic fractionations are limited in igneous systems, except for Eu. More efforts should be focused on low-temperature processes and kinetic effects at high temperatures for REE stable isotopes. Cerium isotopic fractionation is predominantly reported as ^142^Ce/^140^Ce, which is influenced by NFS. Notably, cerium isotopic fractionation has been observed in low-temperature samples associated with processes like seawater adsorption, particularly in ferromanganese oxy/hydroxides. The elucidation of the underlying mechanism calls for an additional investigation.

## Supporting Information Available

Formula for correcting isotope fractionation induced by isobaric interferences (PDF)
