# Supplementary material for: Determination of Rare Earth Element Isotopic Compositions Using Sample-Standard Bracketing and Double-Spike Approaches
Source: ACS Earth Space Chem. 2023 Nov 6;7(11):2222–38. doi: 10.1021/acsearthspacechem.3c00172 (PMC10658628; doi:10.1021/acsearthspacechem.3c00172)
Supplement: Supplementary file 1 — sp3c00172_si_001.pdf [file sp3c00172_si_001.pdf]

# Determination of Rare Earth Element Isotopic Compositions Using Sample-Standard Bracketing and Double-Spike Approaches

*Justin Y. Hu*[†,§,*], *Francois L.H. Tissot*[†,‖], *Reika Yokochi*[†], *Thomas J. Ireland*[†,⊥], *Nicolas Dauphas*[†], *Helen M. Williams*[§].

†Origins Laboratory, Department of the Geophysical Sciences and Enrico Fermi Institute, The University of Chicago, Chicago, IL 60637, USA.

§Department of Earth Sciences, University of Cambridge, Cambridge CB2 3EQ, UK.

‖The Isotoparium, Division of Geological and Planetary Sciences, California Institute of Technology, Pasadena, 1200 E. California Blvd, CA 91125, USA.

⊥Department of Earth and Environment, Boston University, 685 Commonwealth Avenue, Boston, MA 02215.

*jh2363@cam.ac.uk

## Table of Contents

Formula for correcting isotope fractionation induced by isobaric interferences………………S1

## Formula for correcting isotope fractionation induced by isobaric interferences

Below we build on Hu and Dauphas[1] to derive a more comprehensive formula to consider the influence of both isotope anomalies and isobaric interferences on the DS approach.

Take differentials on both sides of Eq. 5,

$$\left[R_{\mathrm{sp}}^{\mathrm{i/k}} - R_{\mathrm{std}}^{\mathrm{i/k}}\left(\frac{m_{\mathrm{i}}}{m_{\mathrm{k}}}\right)^{\alpha}\right]\mathrm{d}f + (1-f)R_{\mathrm{std}}^{\mathrm{i/k}}\left(\frac{m_{\mathrm{i}}}{m_{\mathrm{k}}}\right)^{\alpha}\ln\left(\frac{m_{\mathrm{i}}}{m_{\mathrm{k}}}\right)\mathrm{d}\alpha + \left[(1-f)R_{\mathrm{std}}^{\mathrm{i/k}}\left(\frac{m_{\mathrm{i}}}{m_{\mathrm{k}}}\right)^{\alpha} + fR_{\mathrm{sp}}^{\mathrm{i/k}}\right]\ln\left(\frac{m_{\mathrm{i}}}{m_{\mathrm{k}}}\right)\mathrm{d}\beta = \left(\frac{m_{\mathrm{i}}}{m_{\mathrm{k}}}\right)^{-\beta}\mathrm{d}R_{\mathrm{m}}^{\mathrm{i/k}} - (1-f)\left(\frac{m_{\mathrm{i}}}{m_{\mathrm{k}}}\right)^{\alpha}\mathrm{d}R_{\mathrm{std}}^{\mathrm{i/k}}. \text{ (S1)}$$

Define reduced mass $\mu_{\mathrm{i/j}} = \ln\left(\frac{m_{\mathrm{i}}}{m_{\mathrm{j}}}\right)$ and simplify Eq. S1,

$$\left[\frac{R_{\mathrm{sp}}^{\mathrm{i/k}}}{R_{\mathrm{std}}^{\mathrm{i/k}}}\left(\frac{m_{\mathrm{i}}}{m_{\mathrm{k}}}\right)^{-\alpha} - 1\right]\mathrm{d}f + (1-f)\mu_{\mathrm{i/k}}\mathrm{d}\alpha + \left\{1 + f\left[\frac{R_{\mathrm{sp}}^{\mathrm{i/k}}}{R_{\mathrm{std}}^{\mathrm{i/k}}}\left(\frac{m_{\mathrm{i}}}{m_{\mathrm{k}}}\right)^{-\alpha} - 1\right]\right\}\mu_{\mathrm{i/k}}\mathrm{d}\beta = \left(\frac{m_{\mathrm{i}}}{m_{\mathrm{k}}}\right)^{-\alpha-\beta}\frac{\mathrm{d}R_{\mathrm{m}}^{\mathrm{i/k}}}{R_{\mathrm{std}}^{\mathrm{i/k}}} - (1-f)\frac{\mathrm{d}R_{\mathrm{std}}^{\mathrm{i/k}}}{R_{\mathrm{std}}^{\mathrm{i/k}}}. \text{ (S2)}$$

For isotope anomalies, we adopted the definition in Hu and Dauphas[1],

$$\varepsilon_{\mathrm{i/j}} = 10^{4}\mathrm{d}R_{\mathrm{m}}^{\mathrm{i/j}}/R_{\mathrm{std}}^{\mathrm{i/j}}. \text{ (S3)}$$

We define the isobaric interference on isotope $^{\mathrm{i}}\mathrm{E}$ as,

$$\varphi_{\mathrm{i}} = 10^{4}\mathrm{d}I_{\mathrm{i}}/I_{\mathrm{i}}, \text{ (S4)}$$

where $I_{\mathrm{i}}$ is the intensity of isotope $^{\mathrm{i}}\mathrm{E}$. The variation of measured isotope ratio $^{\mathrm{i}}\mathrm{E}/^{\mathrm{j}}\mathrm{E}$ can be written as,

$$\mathrm{d}R_{\mathrm{m}}^{\mathrm{i/j}} = \frac{I_{\mathrm{i}}+\mathrm{d}I_{\mathrm{i}}}{I_{\mathrm{j}}+\mathrm{d}I_{\mathrm{j}}} - \frac{I_{\mathrm{i}}}{I_{\mathrm{j}}} = \frac{I_{\mathrm{i}}+10^{-4}\varphi_{\mathrm{i}}I_{\mathrm{i}}}{I_{\mathrm{j}}+10^{-4}\varphi_{\mathrm{j}}I_{\mathrm{j}}} - \frac{I_{\mathrm{i}}}{I_{\mathrm{j}}}. \text{ (S5)}$$

Equation S5 can be further simplified,

$$\mathrm{d}R_{\mathrm{m}}^{\mathrm{i/j}} = 10^{-4}\frac{\varphi_{\mathrm{i}}-\varphi_{\mathrm{j}}}{1+10^{-4}\varphi_{\mathrm{j}}}R_{\mathrm{m}}^{\mathrm{i/j}} \approx 10^{-4}(\varphi_{\mathrm{i}}-\varphi_{\mathrm{j}})R_{\mathrm{m}}^{\mathrm{i/j}}. \text{ (S6)}$$

Substituting Eq. S3 and S6 into Eq. S2 gives,

$$\left[\frac{R_{\mathrm{sp}}^{\mathrm{i/k}}}{R_{\mathrm{std}}^{\mathrm{i/k}}}\left(\frac{m_{\mathrm{i}}}{m_{\mathrm{k}}}\right)^{-\alpha} - 1\right]\mathrm{d}f + (1-f)\mu_{\mathrm{i/k}}\mathrm{d}\alpha + \left\{1 + f\left[\frac{R_{\mathrm{sp}}^{\mathrm{i/k}}}{R_{\mathrm{std}}^{\mathrm{i/k}}}\left(\frac{m_{\mathrm{i}}}{m_{\mathrm{k}}}\right)^{-\alpha} - 1\right]\right\}\mu_{\mathrm{i/k}}\mathrm{d}\beta = 10^{-4}(\varphi_{\mathrm{i}}-\varphi_{\mathrm{k}})\left(\frac{m_{\mathrm{i}}}{m_{\mathrm{k}}}\right)^{-\alpha-\beta}\frac{R_{\mathrm{m}}^{\mathrm{i/k}}}{R_{\mathrm{std}}^{\mathrm{i/k}}} - 10^{-4}(1-f)\varepsilon_{\mathrm{i/k}}. \text{ (S7)}$$

Substituting Eq. 5 into Eq. S7, one has,

$$\left[\frac{R_{\mathrm{sp}}^{\mathrm{i/k}}}{R_{\mathrm{std}}^{\mathrm{i/k}}}\left(\frac{m_{\mathrm{i}}}{m_{\mathrm{k}}}\right)^{-\alpha} - 1\right]\mathrm{d}f + (1-f)\mu_{\mathrm{i/k}}\mathrm{d}\alpha + \left\{1 + f\left[\frac{R_{\mathrm{sp}}^{\mathrm{i/k}}}{R_{\mathrm{std}}^{\mathrm{i/k}}}\left(\frac{m_{\mathrm{i}}}{m_{\mathrm{k}}}\right)^{-\alpha} - 1\right]\right\}\mu_{\mathrm{i/k}}\mathrm{d}\beta = 10^{-4}\left\{1 + f\left[\frac{R_{\mathrm{sp}}^{\mathrm{i/k}}}{R_{\mathrm{std}}^{\mathrm{i/k}}}\left(\frac{m_{\mathrm{i}}}{m_{\mathrm{k}}}\right)^{-\alpha} - 1\right]\right\}(\varphi_{\mathrm{i}}-\varphi_{\mathrm{k}}) - 10^{-4}(1-f)\varepsilon_{\mathrm{i/k}}. \text{ (S8)}$$

Since $\left(\frac{m_{\mathrm{i}}}{m_{\mathrm{j}}}\right)^{\alpha} \approx 1$, Eq. S8 can be further approximated to,

$$\left(\frac{R_{\mathrm{sp}}^{\mathrm{i/k}}}{R_{\mathrm{std}}^{\mathrm{i/k}}}-1\right)\mathrm{d}f+(1-f)\mu_{\mathrm{i/k}}\mathrm{d}\alpha+\left[1+f\left(\frac{R_{\mathrm{sp}}^{\mathrm{i/k}}}{R_{\mathrm{std}}^{\mathrm{i/k}}}-1\right)\right]\mu_{\mathrm{i/k}}\mathrm{d}\beta \approx 10^{-4}\left[1+f\left(\frac{R_{\mathrm{sp}}^{\mathrm{i/k}}}{R_{\mathrm{std}}^{\mathrm{i/k}}}-1\right)\right](\varphi_{\mathrm{i}}-\varphi_{\mathrm{k}})-10^{-4}(1-f)\varepsilon_{\mathrm{i/k}}. \text{ (S9)}$$

Define $t_{\mathrm{i/j}}=\frac{R_{\mathrm{sp}}^{\mathrm{i/j}}}{R_{\mathrm{std}}^{\mathrm{i/j}}}-1$ and Eq. S9 can be simplified,

$$t_{\mathrm{i/k}}\mathrm{d}f+(1-f)\mu_{\mathrm{i/k}}\mathrm{d}\alpha+\left(1+ft_{\mathrm{i/k}}\right)\mu_{\mathrm{i/k}}\mathrm{d}\beta=10^{-4}\left[\left(1+ft_{\mathrm{i/k}}\right)(\varphi_{\mathrm{i}}-\varphi_{\mathrm{k}})-(1-f)\varepsilon_{\mathrm{i/k}}\right]. \text{ (S10)}$$

Applying Eq. S10 to three different isotope ratios $^{2}\mathrm{E}/^{1}\mathrm{E}$, $^{3}\mathrm{E}/^{1}\mathrm{E}$, and $^{4}\mathrm{E}/^{1}\mathrm{E}$ gives a set of three linear equations of unknowns $\mathrm{d}f$, $\mathrm{d}\alpha$, and $\mathrm{d}\beta$, which can be solved for $\mathrm{d}\alpha$ using Cramer's rule,

$$\mathrm{d}\alpha=\frac{\begin{vmatrix} t_{2/1} & 10^{-4}\left[\left(1+ft_{2/1}\right)(\varphi_2-\varphi_1)-(1-f)\varepsilon_{2/1}\right] & \left(1+ft_{2/1}\right)\mu_{2/1} \\ t_{3/1} & 10^{-4}\left[\left(1+ft_{3/1}\right)(\varphi_3-\varphi_1)-(1-f)\varepsilon_{3/1}\right] & \left(1+ft_{3/1}\right)\mu_{3/1} \\ t_{4/1} & 10^{-4}\left[\left(1+ft_{4/1}\right)(\varphi_4-\varphi_1)-(1-f)\varepsilon_{4/1}\right] & \left(1+ft_{4/1}\right)\mu_{4/1} \end{vmatrix}}{\begin{vmatrix} t_{2/1} & (1-f)\mu_{2/1} & \left(1+f\,t_{2/1}\right)\mu_{2/1} \\ t_{3/1} & (1-f)\mu_{3/1} & \left(1+f\,t_{3/1}\right)\mu_{3/1} \\ t_{4/1} & (1-f)\mu_{4/1} & \left(1+f\,t_{4/1}\right)\mu_{4/1} \end{vmatrix}}. \text{ (S11)}$$

Equation S11 can be split to give,

$$\mathrm{d}\alpha=10^{-4}\frac{\begin{vmatrix} t_{2/1} & \left(1+ft_{2/1}\right)(\varphi_2-\varphi_1) & \left(1+f\,t_{2/1}\right)\mu_{2/1} \\ t_{3/1} & \left(1+ft_{3/1}\right)(\varphi_3-\varphi_1) & \left(1+f\,t_{3/1}\right)\mu_{3/1} \\ t_{4/1} & \left(1+ft_{4/1}\right)(\varphi_4-\varphi_1) & \left(1+f\,t_{4/1}\right)\mu_{4/1} \end{vmatrix}}{\begin{vmatrix} t_{2/1} & (1-f)\mu_{2/1} & \left(1+f\,t_{2/1}\right)\mu_{2/1} \\ t_{3/1} & (1-f)\mu_{3/1} & \left(1+f\,t_{3/1}\right)\mu_{3/1} \\ t_{4/1} & (1-f)\mu_{4/1} & \left(1+f\,t_{4/1}\right)\mu_{4/1} \end{vmatrix}}+10^{-4}\frac{\begin{vmatrix} t_{2/1} & -(1-f)\varepsilon_{2/1} & \left(1+f\,t_{2/1}\right)\mu_{2/1} \\ t_{3/1} & -(1-f)\varepsilon_{3/1} & \left(1+f\,t_{3/1}\right)\mu_{3/1} \\ t_{4/1} & -(1-f)\varepsilon_{4/1} & \left(1+f\,t_{4/1}\right)\mu_{4/1} \end{vmatrix}}{\begin{vmatrix} t_{2/1} & (1-f)\mu_{2/1} & \left(1+f\,t_{2/1}\right)\mu_{2/1} \\ t_{3/1} & (1-f)\mu_{3/1} & \left(1+f\,t_{3/1}\right)\mu_{3/1} \\ t_{4/1} & (1-f)\mu_{4/1} & \left(1+f\,t_{4/1}\right)\mu_{4/1} \end{vmatrix}}. \text{ (S12)}$$

The second term on the right hand side of Eq. S12 has been derived in Eq. 13-24 in Hu and Dauphas[1], denoted $\mathrm{d}\alpha_{\mathrm{anom}}$ here as isotope fractionation induced by isotope anomalies. We hereby define the first term as $\mathrm{d}\alpha_{\mathrm{intf}}$ for isotope fractionation induced by isobaric interferences. Therefore, we have,

$$\mathrm{d}\alpha=\mathrm{d}\alpha_{\mathrm{anom}}+\mathrm{d}\alpha_{\mathrm{intf}}. \text{ (S13)}$$

Let's define,

$$s_{\mathrm{i/j}}=\frac{fR_{\mathrm{sp}}^{\mathrm{i/j}}-(1-f)R_{\mathrm{std}}^{\mathrm{i/j}}}{R_{\mathrm{std}}^{\mathrm{i/j}}}-1\approx\frac{R_{\mathrm{m}}^{\mathrm{i/j}}}{R_{\mathrm{std}}^{\mathrm{i/j}}}-1. \text{ (S14)}$$

The spike proportion $f$ can be written as,

$$f=\frac{R_{\mathrm{m}}^{\mathrm{i/j}}-R_{\mathrm{std}}^{\mathrm{i/j}}}{R_{\mathrm{sp}}^{\mathrm{i/j}}-R_{\mathrm{std}}^{\mathrm{i/j}}}\approx\frac{s_{\mathrm{i/j}}}{t_{\mathrm{i/j}}}. \text{ (S15)}$$

Substituting Eq. S14 and Eq. S15 into the first moment of Eq. S12, $\mathrm{d}\alpha_{\mathrm{intf}}$ can be simplified,

$$\mathrm{d}\alpha_{\mathrm{intf}}=10^{-4}\frac{\begin{vmatrix}\frac{(1+s_{2/1})(\varphi_2-\varphi_1)}{s_{2/1}} & \frac{(1+s_{3/1})(\varphi_3-\varphi_1)}{s_{3/1}} & \frac{(1+s_{4/1})(\varphi_4-\varphi_1)}{s_{4/1}}\\ (1+s_{2/1})\mu_{2/1} & (1+s_{3/1})\mu_{3/1} & (1+s_{4/1})\mu_{4/1}\end{vmatrix}}{(1-f)\begin{vmatrix}\mu_{2/1} & \mu_{3/1} & \mu_{4/1}\\ s_{2/1} & s_{3/1} & s_{4/1}\\ s_{2/1}\mu_{2/1} & s_{3/1}\mu_{3/1} & s_{4/1}\mu_{4/1}\end{vmatrix}}, \text{ (S16)}$$

The variation in isotope fractionation $\mathrm{d}\delta_{\mathrm{i/j}}^{\mathrm{intf}}=10^{3}\mu_{\mathrm{i/j}}\mathrm{d}\alpha_{\mathrm{intf}}$ can therefore be written as,

$$\mathrm{d}\delta_{\mathrm{i/j}}^{\mathrm{intf}}=\frac{\mu_{\mathrm{i/j}}\begin{vmatrix}\frac{(1+s_{2/1})(\varphi_2-\varphi_1)}{s_{2/1}} & \frac{(1+s_{3/1})(\varphi_3-\varphi_1)}{s_{3/1}} & \frac{(1+s_{4/1})(\varphi_4-\varphi_1)}{s_{4/1}}\\ (1+s_{2/1})\mu_{2/1} & (1+s_{3/1})\mu_{3/1} & (1+s_{4/1})\mu_{4/1}\end{vmatrix}}{10(1-\mathrm{f})\begin{vmatrix}\mu_{2/1} & \mu_{3/1} & \mu_{4/1}\\ s_{2/1} & s_{3/1} & s_{4/1}\\ s_{2/1}\mu_{2/1} & s_{3/1}\mu_{3/1} & s_{4/1}\mu_{4/1}\end{vmatrix}}. \text{ (S17)}$$

Expanding Eq. S17,

$$\mathrm{d}\delta_{\mathrm{i/j}}^{\mathrm{intf}}=\frac{\mu_{\mathrm{i/j}}}{10(1-\mathrm{f})O_1^{1,2,3,4}}\left[N_{2/1}^{1,2,3,4}(\varphi_2-\varphi_1)+N_{3/1}^{1,2,3,4}(\varphi_3-\varphi_1)+N_{4/1}^{1,2,3,4}(\varphi_4-\varphi_1)\right], \text{ (S18)}$$

where,

$$O_1^{1,2,3,4}=s_{3/1}s_{4/1}\mu_{4/3}\mu_{2/1}+s_{2/1}s_{4/1}\mu_{2/4}\mu_{3/1}+s_{2/1}s_{3/1}\mu_{3/2}\mu_{4/1}, \text{ (S19)}$$

$$N_{2/1}^{1,2,3,4}=\left(1+s_{2/1}\right)\left(s_{3/1}\mu_{4/1}-s_{4/1}\mu_{3/1}+s_{4/1}s_{3/1}\mu_{4/3}\right), \text{ (S20)}$$

$$N_{3/1}^{1,2,3,4}=\left(1+s_{3/1}\right)\left(s_{4/1}\mu_{2/1}-s_{2/1}\mu_{4/1}+s_{2/1}s_{4/1}\mu_{2/4}\right), \text{ (S21)}$$

$$N_{4/1}^{1,2,3,4}=\left(1+s_{4/1}\right)\left(s_{2/1}\mu_{3/1}-s_{3/1}\mu_{2/1}+s_{2/1}s_{3/1}\mu_{2/3}\right). \text{ (S22)}$$

REFERENCES

(1) Hu, J. Y.; Dauphas, N. Double-spike data reduction in the presence of isotopic anomalies. *Journal of Analytical Atomic Spectrometry* **2017**, *32* (10), 2024-2033.
